# Association Between Fat Mass to Lean Body Mass Ratio and All-Cause Mortality Among Middle-Aged and Elderly Cancer Patients Without Obesity: A Multi-Center Observational Study in China

**DOI:** 10.3389/fnut.2022.914020

**Published:** 2022-06-16

**Authors:** Hongmei Xue, Hongzhen Du, Ying Xie, Yijing Zhai, Shiming Song, Bin Luo, Hong Qiu, Kunhua Wang, Jiuwei Cui, Chunhua Song, Hongxia Xu, Wei Li, Hanping Shi, Zengning Li, The Investigation on Nutrition Status and Its Clinical Outcome of Common Cancers (INSCOC) Group, Zengqing Guo

**Affiliations:** ^1^Department of Clinical Nutrition, The First Hospital of Hebei Medical University, Shijiazhuang, China; ^2^Hebei Province Key Laboratory of Nutrition and Health, Shijiazhuang, China; ^3^Department of Oncology, Tongji Hospital, Tongji Medical College, Huazhong University of Science and Technology, Wuhan, China; ^4^Department of Gastrointestinal Surgery, Institute of Gastroenterology, The First Affiliated Hospital of Kunming Medical University, Kunming, China; ^5^Cancer Center, The First Hospital of Jilin University, Changchun, China; ^6^Department of Epidemiology, College of Public Health, Zhengzhou University, Zhengzhou, China; ^7^Department of Nutrition, Daping Hospital and Research Institute of Surgery, Third Military Medical University, Chongqing, China; ^8^Department of Gastrointestinal Surgery, Beijing Shijitan Hospital, Capital Medical University, Beijing, China; ^9^Department of Clinical Nutrition, Beijing Shijitan Hospital, Capital Medical University, Chinese Society of Nutritional Oncology, Beijing, China

**Keywords:** fat mass to lean body mass ratio, percentage of body fat, fat mass, all-cause mortality, elderly cancer patients

## Abstract

**Objective:**

We aimed to investigate the association between fat mass to lean body mass ratio (RFL), percentage of body fat (PBF), and fat mass (FM) with mortality among middle-aged and elderly cancer patients without obesity.

**Methods:**

This prospective hospital-based cohort study comprised 3,201 patients with stage I to IV cancer aged 40 years or above (mean age: 58 years for female patients and 61 years for male patients; mean length of follow-up was 1.67 years; the maximal follow-up length was 6.42 years). FM and PBF were measured by bioelectrical impedance analysis (BIA). Cox proportional hazard models were used, and adjusted hazard ratios (HRs) were estimated.

**Results:**

We revealed a significant association between RFL and all-cause mortality among men aged ≥60 years after adjusting for confounders. Compared with those in the lowest tertile of RFL, elderly men in the medium and highest tertile had a 35 and 34% lower hazard of death from any cause, respectively. After additionally adjusted for C-reaction protein (CRP), HRs of medium and high tertile of RFL became short of statistical significance [medium tertile: adjusted HRs (95% CI) = 0.74 (0.46, 1.20); highest tertile: adjusted HRs (95% CI) = 0.84 (0.53, 1.33)]. Among elderly women, RFL was significantly related to all-cause mortality only when the additional adjustment for CRP [medium tertile: adjusted HRs (95% CI) = 2.08 (1.08, 4.01); highest tertile: adjusted HRs (95% CI) = 0.90 (0.45, 1.81)]. No significant association between RFL and all-cause mortality was observed among female participants or male participants aged less than 60 years.

**Conclusion:**

Our findings showed a significant non-linear association between RFL and all-cause mortality, which was observed only in elderly men, and might be attenuated by their inflammation state.

## Introduction

Increasing obesity rates remain a substantial public health and clinical concern in China and around the world (1). In China, over one in seven individuals meets the criteria for overall obesity, and one in three individuals meets the criteria for abdominal obesity (2). Body mass index (BMI) is known as the most commonly used measure of body adipose (3), being reported by many epidemiological studies as a significant factor for increased risk of many chronic diseases and mortality (4–8). However, the association between BMI and mortality is controversial, with various types of J-shaped, U-shaped, and linear relations between BMI and mortality being found (9). The reason for this is that body composition index like fat and muscle and their interaction with all-cause mortality does not properly be considered.

Both lean body mass (LBM) and fat mass (FM) have been shown to be related to survival in some cancer patients (10–18). Studies focused on LBM suggested that loss of muscle mass or fat-free mass is detrimental to the prognosis of cancer patients (10–16), while conclusions for adipose tissue have been inconsistent. The prospective studies on Paris middle-aged men followed up for 15 years (17) and the Netherlands patients (18) indicate that increased risk of cancer death is associated with lower subcutaneous fat (17) or early fat mass loss during chemoradiotherapy (18). Two other prospective population-based studies in the United States found a strong positive monotonic association between fat mass and all-cause mortality among men (10) and breast cancer-specific death among black women (11). Association between fat mass and all-cause mortality needs to be studied. Balance of fat mass to lean body mass is essential for human health, including non-alcoholic fatty liver disease, type 2 diabetes mellitus, metabolic syndrome, and arterial stiffness (19–21). To the best of our knowledge, studies on the effects of fat mass to lean body mass ratio (RFL) on cancer survival are scarce and also need to be further explored, because the balance of fat mass and lean body mass may be more important for the prognosis of cancer than the fat mass or muscle alone (22).

Considering the limited research, we aimed to explore the relationship between RFL, percentage of body fat (PBF), FM, and LBM for all-cause mortality among cancer patients aged 40 years or older from the Investigation on Nutrition Status and Its Clinical Outcome of Common Cancers (INSCOC), the results of which are expected to provide ideas and directions for improving the prognosis of cancer patients.

## Materials and Methods

### Study Population

We used data from the INSCOC project, an observational, multi-centered, and hospital-based prospective cohort study that aims to determine the prevalence of malnutrition in cancer patients in China and its relationship to physical performance and quality of life. Details of the study protocol have been described elsewhere (23). Briefly, patients diagnosed with malignant tumors based on the anatomical site code of the tenth edition of the International Classification of Disease (ICD-10) were recruited by clinical investigators in various departments of the participating hospitals. All patients were regularly followed up by telephone interviews or outpatient visits. All of the participants signed an informed consent form. The study protocol and procedures were approved by the INSCOC Research Ethics Committee and the Institutional Review Boards of all participating institutions (The First Hospital of Hebei Medical University, Tongji Hospital, The First Affiliated Hospital of Kunming Medical University, the First Hospital of Jilin University, Zhengzhou University, and Beijing Shijitan Hospital), and the study has been registered in Chinese Clinical Trial Register (URL)^1^ (Register No: ChiCTR1800020329).

**FIGURE 1:**
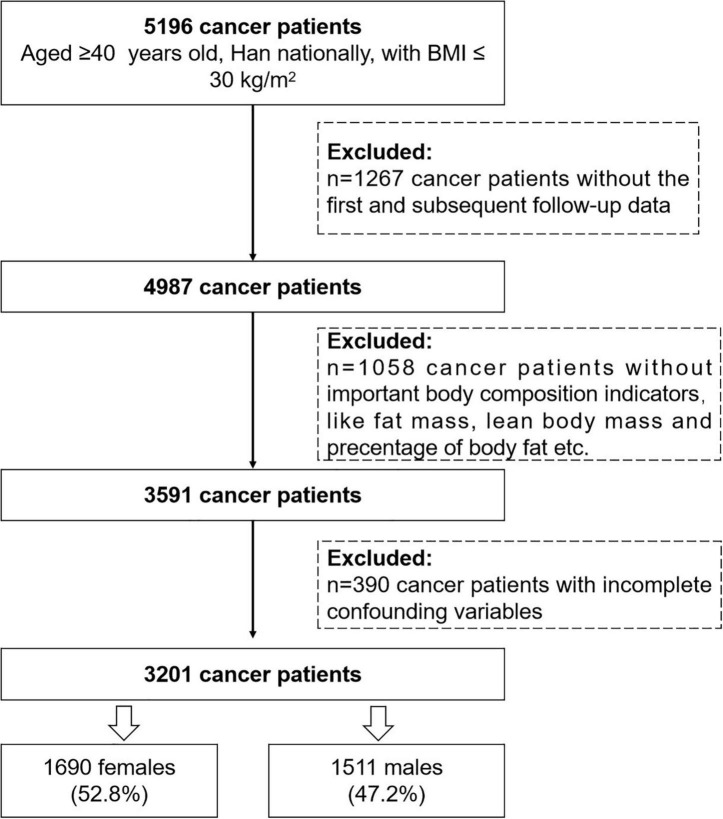
Flowchart for the study sample.

Data used in this analysis were identified from the INSCOC project between 2014 January and 2021 May. Initially, 5,196 patients aged ≥40 years, Han nationally, and BMI ≤ 30 kg/m^2^ were included in our analysis. Of these, 4,987 patients had at least 1 year follow-up assessment by the end of May 2021. Since we are interested in the effect of body composition on mortality, 1,058 cancer patients without important body composition indicators like FM, LBM, PBF, and RFL were excluded, and 390 participants with incomplete information on potential confounders were further excluded. Finally, 3,201 cancer patients (1,690 female patients, 52.8%; 1,511 male patients, 47.2%) were eligible for this analysis (Figure 1).

### Assessment of Exposure Variable and Covariates

Anthropometric measurements were performed by trained medical workers according to the standard procedures at baseline. Body weight and height were measured in light indoor clothing without shoes to the nearest 0.1 kg and 0.1 cm, respectively. Body mass index (BMI) is then calculated as weight (kg)/height (m)^2^. The handgrip strength (HGS, non-dominant hand) is measured by an electronic handgrip dynamometer. Weight, height, and HGS were each averaged on the basis of two measurements. Participants were also assessed with a multi-frequency bioelectrical impedance analysis (InBodyS10; Bio-space, Tokyo, Japan; DBA-A50, China). With this method, PBF, FM, and LBM are automatically and simultaneously obtained, and RFL was calculated.

For each patient enrolled in this study, a printed questionnaire was scheduled for them, including:

a) *Basic information and tumor characteristics*: Sex; birth date; nationality; residence (urban/rural); education; occupation; marital status; healthcare type; commodities like diabetes mellitus, hypertension, coronary heart disease, and cirrhosis; cancer types; family history of cancer; and previous treatments (surgery, chemotherapy, and radiotherapy).

b) *Lifestyle*: Like smoking (current, former, and never), alcohol drinking (yes/no), and tea drinking (yes/no).

c) *Nutritional risk assessment*: Nutritional risk screening 2002 (NRS 2002) (24), patient generated-subjective global assessment (PG-SGA) (25), and Karnofsky performance status (KPS) (26) were used to assess cancer patients’ physical status.

d) *Quality of life*: This was assessed by the European Organization for Research and Treatment of Cancer (EORTC) QLQ-C30 Version 3 (27).

e) Additionally, nutritional interventions [parenteral nutrition (PN) or enteral nutrition (EN) intervention], duration of hospital stay, and anticancer treatments were collected, and C-reaction protein (CRP) was also tested at baseline.

### Ascertainment of Outcomes

In the follow-up period, data on weight, BMI, KPS, quality of life, date of death, and death from cancer or other causes (traffic accidents or cardiovascular diseases or unknown if the relatives refused to answer) will be assessed at 30 days and at 1, 2, 3, 4, and 5 years after recruitment (23).

In this analysis, we considered survival time and all-cause mortality (cancer or other causes) as the outcome variables. Survival time was calculated based on the time difference between the baseline investigated time and date of death or date of the last follow-up for whom were lost to follow-up.

### Statistical Analysis

The SAS procedures (SAS, version 9.4, SAS Institute Inc., Cary, NC, United States) were used for data analyses. All analyses were performed with a significance level at *p* < 0.05, except for interaction tests, where *p* < 0.1 was considered significant. Normality of all continuous variables was examined by using normal probability plots and the Kolmogorov–Smirnov test. Given their non-normality, all continuous variables were presented as median (25th percentile, 75th percentile). Preliminary analyses indicated sex and age interactions between the relation of RFL and mortality (sex: *p* for interaction = 0.019; age: *p* for interaction = 0.013). Therefore, we stratified all subsequent analyses by sex and age (<60 years and ≥60 years). Spearman’s correlations were used first to assess the relations between NRS 2002 score and PG-SGA in this analysis. Since the correlation coefficient was 0.38 for NRS 2002 score and PG-SGA, two of them could not be considered concurrently as potential confounding factors. We grouped the independent continuous covariates (RFL, PBF, and FM) into tertiles for illustration of their association with general characteristics and time to mortality. Significant differences for non-normally distributed continuous variables were analyzed by Kruskal–Wallis tests, and chi-square tests were used for categorical variables.

The univariate associations between the study subgroups and survival were evaluated using Kaplan–Meier curves and log-rank tests. Cox proportional hazard models were used to investigate the association between RFL, PBF, FM, and mortality. The results are shown as hazard ratios (HRs) together with 95% confidence intervals. The Kaplan–Meier curves and log-rank tests were used to visually and statistically estimate the proportional hazards assumption. In the unadjusted models, the correlation analyses between time to mortality and RFL, PBF, and FM were carried out first. In a further step, potential covariates that may affect these associations were added, which included age (years), education level (≤12 years and >12 years of schooling), smoking (current, smoke ever, never smoking), alcohol drinking (yes or no), family history of cancer (yes or no), commodities (yes or no), nutrition support (PN, EN or no), KPS, duration of hospital stays, NRS 2002 score (<3, ≥3), cancer types (lung cancer, breast cancer, colorectal cancer, esophageal cancer, gastric cancer, and others), cancer TNM stage I to IV, previous treatments (surgery, chemotherapy, radiotherapy, others), handgrip strength, and CRP. Each variable was initially considered separately: only variables that had their own independent significant effect in the univariate models or that substantially modified the association were included in the subsequent multivariable analyses (28) (Supplementary Tables 2, 3). In the regress analysis, the covariables models adjusted were as follows: Model A: adjusted for age, education, smoking, alcohol drinking, family history of cancer, version of Body Composition Analyzer; Model B: as model A and additionally adjusted for Karnofsky performance scores, duration of hospital stays, NRS 2002 scores, nutrition support, and handgrip strength; Model C: as Model B and additionally adjusted for commodities, previous treatments, cancer types, TNM stages, and quality of life score; Model D: as Model C and additionally adjusted CRP. Additionally, we conducted some sensitive analyses that aimed to assess whether the association between RFL, FM, or LBM and all-cause mortality was independent of each other (for RFL, LBM was adjusted), and three of them were independent of BMI based on the correlation between body composition indicators (Supplementary Tables 5–9).

## Results

Of the 3,201 patients recruited in our analysis, nearly half of our participants were female patients (52.8%). The median age was 59.39 years, and 1,677 participants were aged ≥60 years. Table 1 shows the baseline characteristics of the sample in this study according to tertiles of RFL. People in higher tertile of RFL were older, had higher education level, and smoked more. Fat mass increased with higher RFL. Cancer patients with higher RFL tend to have higher BMI, higher PBF, and higher KPS. Moreover, handgrip strength increased in the medium tertile of RFL and slightly decreased with higher RFL. However, patients in the highest tertile of RFL had lower mortality rates, life quality, and NRS 2002 scores compared with that in the lowest tertile (all *p*-values < 0.02).

**TABLE 1:** Characteristics^a^ of participants stratified by tertile of fat mass to lean body mass ratio.

	RFL			*p*
	T1	T2	T3	
RFL	0.24 (0.18, 0.35)	0.44 (0.30, 0.51)	0.59 (0.43, 0.68)	–
n (%)	1066 (33.30)	1068 (33.36)	1067 (33.33)	–
Female (%)	563 (52.81)	564 (52.81)	563 (52.76)	1.000
Age (yrs)	57.89 (50.72, 64.33)	59.22 (52.22, 65.09)	61.00 (54.59, 67.06)	<0.001
Mortality (%)	283 (26.55)	226 (21.16)	239 (22.40)	0.009
Quality of life	47 (42, 53)	46 (42, 52)	46 (42, 52)	0.009
**Nutritional indices**				
Percentage of body fat (%)	18.20 (14.50, 24.90)	29.10 (21.80, 32.35)	35.80 (28.70, 38.80)	<0.001
Fat mass (kg)	10.44 (8.12, 13.35)	16.72 (14.56, 19.32)	23.63 (20.25, 26.94)	<0.001
Lean body mass(kg)	42.10 (37.40, 48.00)	43.00 (37.50, 49.65)	42.30 (36.70, 48.90)	0.117
Body mass index (kg/m^2^)	20.59 (19.14, 22.21)	23.51 (21.97, 24.88)	25.85 (24.22, 27.82)	<0.001
Handgrip strength	22.52 (17.40, 30.20)	23.80 (18.40, 31.85)	23.70 (18.00, 31.00)	0.020
C-reactive protein	3.29 (3.02, 18.60)	3.23 (2.31, 13.22)	3.79 (3.00, 13.10)	0.154
PG-SGA				
0–1	422 (13.18)	525 (16.40)	560 (17.49)	<0.001
2–8	528 (16.49)	423 (14.46)	469 (13.40)	
≥9	116 (3.62)	80 (2.50)	78 (2.44)	
NRS2002 scores (≥ 3,%)	257 (24.11)	119 (11.14)	96 (9.00)	<0.001
KPS scores	90 (90, 90)	90 (90, 100)	90 (90, 100)	0.009
Nutrition support ^b^ (yes,%)	220 (20.64)	197 (18.45)	183 (17.15)	0.114
**Cancer types**				
Lung cancer	365 (11.40)	368 (11.50)	355 (11.09)	<0.001
Breast cancer	166 (5.19)	226 (7.06)	287 (8.97)	
Colorectal cancer	125 (3.91)	170 (5.31)	149 (4.65)	
Esophageal cancer	39 (1.22)	24 (0.75)	14 (0.44)	
Gastric cancer	32 (1.00)	40 (1.25)	46 (1.44)	
Others	339 (10.59)	240 (7.50)	216 (6.75)	
**TNM stages**				
I	99 (4.01)	105 (4.25)	106 (4.29)	0.888
II	158 (6.40)	151 (6.12)	137 (5.55)	
III	213 (8.63)	209 (8.46)	228 (9.23)	
IV	164 (6.64)	340 (13.78)	367 (14.87)	
**Treatments**				
Surgery	207 (6.47)	178 (5.56)	187 (5.84)	0.001
Chemotherapy	653 (20.40)	652 (20.37)	600 (18.74)	
Radiotherapy	21 (0.66)	17 (0.53)	40 (1.25)	
Others	185 (5.78)	221 (6.90)	240 (7.50)	
**Duration of hospital stays (days)**	11.00 (7.00, 19.00)	11.00 (7.00, 18.00)	11.00 (7.00, 18.00)	0.736
**Socio-demographics**				
High education level ^c^ (%)	751 (70.45)	823 (77.06)	814 (76.29)	0.001
Smoking (Current,%)	316 (29.64)	253 (23.69)	249 (23.34)	0.003
Alcohol drinking (yes,%)	204 (19.14)	192 (17.98)	195 (18.28)	0.774
Tea (yes,%)	179 (17.26)	207 (19.38)	211 (19.78)	0.158
Family history of cancer (yes,%)	168 (15.76)	213 (19.94)	192 (17.99)	0.042
Comorbidity (yes,%)	391 (36.68)	330 (30.90)	266 (24.93)	<0.001

*PG-SGA, patient-generated subjective nutrition assessment; NRS 2002, nutrition risk screening 2002; KPS, Karnofsky performance scores; RFL, ratio of fat mass to lean body mass.*

*^a^Values are medians (Q1, Q3) or frequencies. Test for difference between tertiles of fat mass to lean body mass ratio was performed by using Kruskal–Wallis tests for non-normally distributed continuous variables and chi-square test for categorical variables.*

*^b^Receive enteral nutrition or parenteral nutrition.*

*^c^School years ≥ 12 years.*

During the follow-up period, we identified 748 deaths. Association between RFL and all-cause mortality among cancer patients stratified by sex and age using Cox proportional hazard regression models is presented in Table 2. After adjusted for age, education, smoking, alcohol drinking, family history of cancer, BIA models, KPS, duration of hospital stays, NRS 2002 score, nutrition support, handgrip strength, commodities, previous treatments, cancer types, and quality of life score, a non-linear association between RFL and all-cause mortality among men aged ≥60 years was revealed. Compared with those in the lowest tertile of RFL, elderly men in the medium and highest tertile had a 35 and 34% lower hazard of death from any cause, respectively [medium: adjusted HRs (95% CI) = 0.65 (0.45, 0.94); high: adjusted HRs (95% CI) = 0.66 (0.46, 0.95)]. After additionally adjusted for CRP, HRs of medium and high tertile of RFL became short of statistical significance [medium tertile: adjusted HRs (95% CI) = 0.74 (0.46, 1.20); highest tertile: adjusted HRs (95% CI) = 0.84 (0.53, 1.33)]. Similar trends were observed when we take the medium tertile as the reference (Supplementary Table 4). In the sensitivity analysis, BMI or LBM was put into the adjusted model (Supplementary Table 8), and we found that the non-linear association between RFL and all-cause mortality remained significant. Among elderly women, RFL was significantly related to all-cause mortality only when the additional adjustment for CRP [medium tertile: adjusted HRs (95% CI) = 2.08 (1.08, 4.01); highest tertile: adjusted HRs (95% CI) = 0.90 (0.45, 1.81)], which showed that medium tertile of RFL may be a risk factor after the inflammatory state was considered. No significant associations were observed among cancer patients aged less than 60 years.

**TABLE 2:** Association between fat mass to lean body mass ratio and all-cause mortality using Cox proportional hazard regression.^a^

	<60 years			P for trend	≥60 years			P for trend
	T1	T2	T3		T1	T2	T3	
**Female**								
Unadjusted model	1	0.77 (0.53, 1.11)	0.72 (0.49, 1.06)	0.172	1	0.84 (0.57, 1.24)	0.74 (0.51, 1.07)	0.282
Model A^b^	1	0.79 (0.55, 1.15)	0.69 (0.47, 1.02)	0.159	1	0.95 (0.64, 1.41)	0.83 (0.57, 1.22)	0.624
Model B^b^	1	0.99 (0.67, 1.46)	0.84 (0.56, 1.26)	0.661	1	1.07 (0.71, 1.61)	0.96 (0.64, 1.44)	0.857
Model C^b^	1	1.04 (0.66, 1.64)	0.95 (0.57, 1.56)	0.935	1	1.30 (0.82, 2.07)	1.10 (0.69, 1.75)	0.521
Model D^b^	1	0.86 (0.48, 1.54)	0.66 (0.34, 1.30)	0.486	1	2.08 (1.08, 4.01)	0.90 (0.45, 1.81)	0.018
**Male**								
Unadjusted model	1	0.83 (0.59, 1.18)	0.87 (0.60, 1.25)	0.549	1	0.58 (0.42, 0.79)	0.66 (0.50, 0.88)	0.001
Model A^b^	1	0.84 (0.59, 1.20)	0.88 (0.61, 1.28)	0.610	1	0.58 (0.43, 0.80)	0.63 (0.47, 0.84)	0.001
Model B^b^	1	0.86 (0.60, 1.23)	0.99 (0.67, 1.44)	0.682	1	0.57 (0.42, 0.79)	0.62 (0.46, 0.84)	0.001
Model C^b^	1	0.80 (0.51, 1.27)	1.00 (0.64, 1.57)	0.569	1	0.65 (0.45, 0.94)	0.66 (0.46, 0.95)	0.033
Model D^b^	1	0.98 (0.54, 1.78)	1.25 (0.72, 2.17)	0.653	1	0.74 (0.46, 1.20)	0.84 (0.53, 1.33)	0.475

*^a^Values are models adjusted HR and 95% confidence interval, ranges for tertiles (T) 1 through 3. Linear trends (p for trend) were obtained with the ratio of fat mass to lean body mass as continuous variables.*

*^b^Model A: adjusted for age, education, smoking, alcohol drinking, family history of cancer, version of Body Composition Analyzer; Model B: as model A and additionally adjusted for Karnofsky performance scores, duration of hospital stays, NRS 2002 score, nutrition support, and handgrip strength; Model C: as Model B and additionally adjusted for commodities, previous treatments, cancer types, TNM stages, and quality of life; Model D: as Model C and additionally adjusted C-reaction protein.*

**FIGURE 2:**
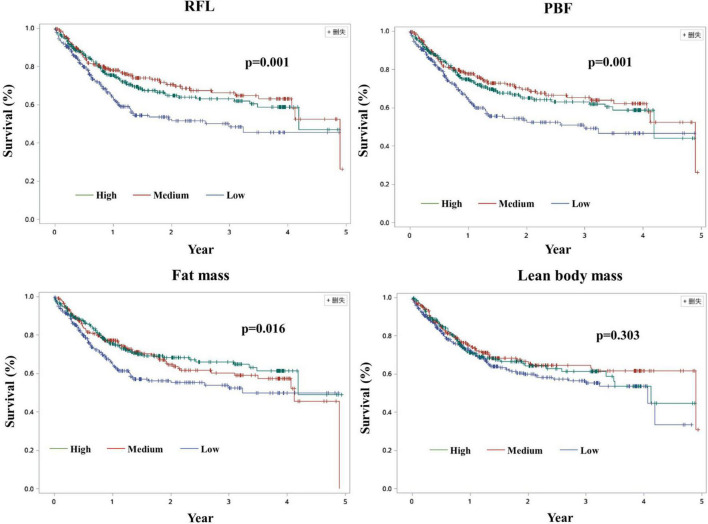
Kaplan–Meier curves among men cancer patients aged ≥60 years. RFL: Fat mass to lean body mass ratio; PBF, Percentage of body fat.

Significant associations were also found between PBF and fat mass and all-cause mortality when we adjusted for age, education, smoking, alcohol drinking, family history of cancer, BIA models, KPS, duration of hospital stays, NRS 2002 score, nutrition support, and handgrip strength (Tables 3, 4). However, the associations became non-significant when commodities, previous treatments, cancer types, TNM stages, and quality of life score were further adjusted. These results changed when we additionally adjusted BMI or LBM in the model of PBF and mortality (Supplementary Table 9). Unlike these, non-significant association between lean body mass and all-cause mortality was observed in the unadjusted and adjusted models (Supplementary Figure 1 and Supplementary Table 1). Additionally, in the sensitivity analysis, the association between FM and all-cause mortality remained even if we adjusted BMI or LBM (Supplementary Table 6). The model of LBM and mortality was sensitive for PBF and FM (Supplementary Table 7). Kaplan–Meier (K-M) curves and log-rank tests for selected body composition variables indicated that patients in the medium tertile of RFL and PBF and patients in the high tertile of FM have a high survival rate among elderly men within limits (Figure 2). Besides, there were some cross of K-M curves of three groups. K-M curves for men aged ≥60 years and women were presented in Supplementary Figures 1–3, respectively. Restricted cubic spline Cox regression between RFL and all-cause mortality among male patients aged ≥60 years was presented in Supplementary Figure 4, which shows that RFL was associated with all cause-mortality among elderly men within limits.

**TABLE 3:** Association between the percentage of body fat and all-cause mortality using Cox proportional hazard regression.

	<60 years			P for trend	≥60 years			P for trend
	T1	T2	T3		T1	T2	T3	
**Female**								
Unadjusted model	1	0.81 (0.56, 1.16)	0.70 (0.47, 1.03)	0.176	1	0.83 (0.56, 1.22)	0.72 (0.50, 1.05)	0.232
Model A^b^	1	0.83 (0.57, 1.20)	0.67 (0.45,1.00)	0.144	1	0.94 (0.63, 1.39)	0.82 (0.56, 1.21)	0.583
Model B^b^	1	1.03 (0.70, 1.51)	0.81 (0.54, 1.23)	0.496	1	1.05 (0.70, 1.59)	0.95 (0.63, 1.42)	0.855
Model C^b^	1	1.07 (0.68, 1.69)	0.88 (0.53, 1.46)	0.746	1	1.22 (0.77, 1.93)	1.07 (0.67, 1.70)	0.695
Model D^b^	1	0.93 (0.53, 1.64)	0.65 (0.33, 1.28)	0.451	1	1.94 (1.01, 3.73)	0.86 (0.43, 1.72)	0.027
**Male**								
Unadjusted model	1	0.83 (0.58, 1.18)	0.88 (0.61, 1.27)	0.563	1	0.61 (0.45, 0.83)	0.68 (0.51, 0.91)	0.003
Model A*^b^*	1	0.84 (0.59, 1.20)	0.88 (0.61, 1.28)	0.608	1	0.61 (0.45, 0.83)	0.64 (0.48, 0.86)	0.002
Model B^b^	1	0.85 (0.60, 1.23)	0.97 (0.66, 1.41)	0.673	1	0.59 (0.43, 0.81)	0.62 (0.46, 0.85)	0.001
Model C^b^	1	0.75 (0.48, 1.18)	0.96 (0.61, 1.50)	0.431	1	0.70 (0.49, 1.01)	0.70 (0.49, 1.00)	0.082
Model D^b^	1	0.89 (0.50, 1.61)	1.21 (0.69, 2.10)	0.595	1	0.83 (0.52, 1.33)	0.91 (0.57, 1.44)	0.745

*^a^Values are models adjusted HR and 95% confidence interval, ranges for tertiles (T) 1 through 3. Linear trends (p for trend) were obtained with the percentage of body fat as continuous variables.*

*^b^Model A: adjusted for age, education, smoking, alcohol drinking, family history of cancer, version of Body Composition Analyzer; Model B: as model A and additionally adjusted for Karnofsky performance scores, duration of hospital stays, NRS 2002 score, nutrition support, and handgrip strength; Model C: as Model B and additionally adjusted for commodities, previous treatments, cancer types, TNM stages, and quality of life; Model D: as Model C and additionally adjusted C-reaction protein.*

**TABLE 4:** Association between fat mass and all-cause mortality using Cox proportional hazard regression.

	<60 years			P for trend	≥60 years			P for trend
	T1	T2	T3		T1	T2	T3	
**Female**								
Unadjusted model	1	0.62 (0.42, 0.90)	0.62 (0.42, 0.90)	0.012	1	0.89 (0.61, 1.30)	0.74 (0.50, 1.08)	0.276
Model A^b^	1	0.62 (0.42, 0.90)	0.61 (0.41, 0.89)	0.010	1	1.01 (0.69, 1.49)	0.88 (0.59, 1.30)	0.725
Model B^b^	1	0.74 (0.50, 1.10)	0.74 (0.49, 1.11)	0.217	1	1.23 (0.81, 1.85)	1.05 (0.70, 1.58)	0.579
Model C^b^	1	0.90 (0.56, 1.44)	0.89 (0.55, 1.45)	0.866	1	1.28 (0.79, 2.07)	1.27 (0.79, 2.02)	0.528
Model D^b^	1	0.83 (0.44, 1.55)	0.69 (0.37, 1.30)	0.513	1	1.82 (0.98, 3.39)	0.95 (0.47, 1.92)	0.062
**Male**								
Unadjusted model	1	0.94 (0.66, 1.35)	0.90 (0.62, 1.30)	0.846	1	0.73 (0.54, 0.98)	0.67 (0.49, 0.90)	0.017
Model A^b^	1	0.94 (0.65, 1.34)	0.91 (0.63, 1.32)	0.877	1	0.71 (0.53, 0.97)	0.63 (0.47, 0.86)	0.009
Model B^b^	1	0.99 (0.68, 1.43)	1.04 (0.71, 1.53)	0.961	1	0.68 (0.50, 0.92)	0.63 (0.46, 0.87)	0.009
Model C^b^	1	1.13 (0.71, 1.80)	1.14 (0.72, 1.81)	0.824	1	0.71 (0.49, 1.01)	0.70 (0.49, 1.02)	0.097
Model D^b^	1	1.13 (0.62, 2.05)	1.33 (0.76, 2.34)	0.601	1	0.98 (0.62, 1.55)	0.91 (0.57, 1.45)	0.906

*^a^Values are models adjusted HR and 95% confidence interval, ranges for tertiles (T) 1 through 3. Linear trends (p for trend) were obtained with fat mass as continuous variables.*

*^b^Model A: adjusted for age, education, smoking, alcohol drinking, family history of cancer, version of Body Composition Analyzer; Model B: as model A and additionally adjusted for Karnofsky performance scores, duration of hospital stays, NRS 2002 score, nutrition support, and handgrip strength; Model C: as Model B and additionally adjusted for commodities, previous treatments, cancer types, TNM stages, and quality of life; Model D: as Model C and additionally adjusted C-reaction protein.*

## Discussion

We found that a significant non-linear association between RFL and all-cause mortality among cancer patients, which was observed only in elderly men, might be attenuated after adjusting for CRP. Body composition indicators among elderly men and women were influenced by their inflammatory state.

Fat mass and lean body mass may act differently on health outcomes including mortality, which have long been used to explain the existence of the “obesity paradox” controversy. The health benefits of a certain amount of adipose tissue are beneficial, and excessive fat may threaten human health. Muscle tissue interacts with adipose tissue. The balance of fat mass and muscle tissue may have important effects on human health. To the best of our knowledge, few studies have focused on the ratio of fat and lean body mass. Some studies have focused on the skeletal muscle mass to visceral fat area ratio and have found a close association between the skeletal muscle mass to visceral fat area ratio and cardiometabolic diseases, including non-alcoholic fatty liver disease, type 2 diabetes mellitus, metabolic syndrome, and arterial stiffness, independent of conventional obesity measures (19–21). For cancer patients, maintaining the right amount of fat is of equal importance to prevent muscle loss. In our study, we calculated RFL and found a significant association between RFL and all-cause mortality among elderly men. Our findings are of important significance for guiding clinical diagnosis and treatment.

Emerging research suggests that the accumulation of fat may be associated with several adverse health outcomes (29–31), including bone mineral density (29, 30), (cardio)metabolic diseases (31), and the prognosis of cancer patients (32). Prospective studies, to date, come to inconsistent conclusions about the fat mass and cancer survival: two studies on Paris middle-aged men (17) and the Netherlands patients (18) to support the accumulation of certain fat are beneficial for the prognosis of cancer patients (17, 18), and two other prospective population-based studies focused on the United States men (10) and black women (11) found a strong positive monotonic association between fat mass and all-cause mortality (10) and breast cancer-specific death (11). In this analysis, we found that an association between fat mass and all-cause mortality was unimpeded by general demographic characteristics, nutriture, and physical fitness indicators, but were affected by commodities, previous treatments, cancer types, TNM stages, quality of life score, and CRP. Combined with the results of RFL and mortality, we could conclude that composite index RFL might better reflect the effect of body composition on the mortality of cancer patients.

The CRP is an immune-inflammatory parameter (33) being reported to directly involve in cardiovascular diseases such as inflammation and atherosclerosis and is the strongest predictor and risk factor of cardiovascular diseases and cancer (34). Cancer patients are in an inflammatory state all the time (35, 36). In this study, the CRP level of the cancer patients ranged widely (0–84.7 mg/L), and the median CRP of the study population was less than 3.37 mg/L. The reasons for this situation may lie in that most of the study population have a history of anti-inflammatory-related treatment. Briefly, considering the importance of inflammation in cancer patients, we additionally adjusted CRP in the final model. In this study, we found that the association between RFL, PBF, and fat mass, and even lean body mass for all-cause mortality was attenuated, which may be inferred that the association between RFL and mortality was affected by CRP to some extent. These results indicated that the inflammatory state may mitigate the benefits of fat. But it is always good to maintain a certain amount of fat. We found that a moderate level of RFL might be a risk factor, and an invertedly U-shaped association was observed between RFL and all-cause mortality. The reasons for this result and the difference between male and female patients should be further studied. We suspect that high FM, low LBM, low level of CRP, and so on might contribute to this phenomenon. For cancer patients, what level of fat should be maintained is very important. More studies on the optimal of fat mass and fat mass to lean body mass among cancer patients are needed.

Fat tissue is an important endocrine system and the largest “energy reservoir” in the body. A number of possible physiological mechanisms underlying the association of body fat mass with cancer progression have been proposed, including circulating insulin and/or insulin-like growth factor 1, altered adipokine levels (i.e., increased leptin and decreased adiponectin), and systemic and tissue-level inflammation (36, 37). Adipose tissue gradually increases with age, including intramuscular fat infiltration and abdominal fat accumulation. But for cancer patients, the amount of fat decreases as the disease progresses. At present, there is a lack of large-scale and multi-regional data on the fat mass of the elderly in China. Further research is needed to determine the range of normal reference values for fat levels in elderly cancer patients, and we should pay more attention to balance fat and muscle. Previous studies have studied the association between muscle loss and adverse health outcomes (38), such as physical disability (39), poorer quality of life (40, 41), and mortality (10–16), which denotes the importance of muscle in the healthcare for older people. Suboptimal LBM may be associated with increased mortality. Non-significant association between lean body mass and all-cause mortality was found in this study, which is because we covered a wide range of cancers (18 cancers) and the effects of muscle mass on different cancers vary. In this study, we detected the lean body mass just at baseline. The absence of muscle tissue changes may also be one of the reasons we get meaningless results. Fat and muscle are not independent of each other. Increasing evidence seems to indicate that most existing chronic non-infectious diseases are affected by both fat and muscle tissue. The association of fat mass and all-cause was significant after further adjustment for LBM may lie in the interactions of muscle, fat, and bone tissues on a cellular level considering their endocrine features. Further investigation focused on the change in body composition and mortality should be conducted.

This study has several strengths. This prospective analysis of a cohort of cancer survivors from multiple geographic areas was specifically designed to assess the association of obesity with survivorship among middle-aged and elder men and women after a diagnosis of cancer. The repeated detailed measurements in participants in conjunction with the ability to adjust for a number of major potential confounders like demographic, health and lifestyle covariates, cancer-related information, and patients’ inflammatory state were the considerable strengths.

However, some limitations of our study should be mentioned. First, data on potential confounders, such as diet were not collected, so we could not exclude the potential confounding effects. Additionally, physical fitness is an important factor for the relationship of body composition and all-cause mortality. Unadjustment of physical fitness was the main limitation of our analysis even though we adjusted the handgrip strength, an indicator of fitness, and some other confounding factors. Considering that BMI has an important impact on the association between RFL and all-cause mortality among cancer patients, and the number of cancer patients with BMI ≥ 30 kg/m^2^ was too small. So, we limited our patients only to BMI ≤ 30 kg/m^2^. Therefore, the external validity of the results, the problem of excluding people with missing data (i.e., selection bias), the limited number of included participants, and the small follow-up time for a prospective cohort study influence the extrapolation of the conclusion. The rate of lost to follow-up was relatively high in this study, which may affect the accuracy of our statistical results. Considering the slightly high rate of lost to follow-up (24.4% vs. 20%), we think our research results are of certain reference significance. The use of bioelectrical impedance analysis (BIA) was another limitation of our study. Bioimpedance is known to be a method that is not the gold standard for assessing fat mass because it presents significantly large variations according to the time of day of the examination and the hydration status of the person assessed, indicating values of fat mass that are not truly presenting great bias and error due to the variation of the total body water content. But considering the gold standard method, Dule Energy X-Ray Absorptiometry (DEXA), which is a relatively large volume and not easy to move, we used BIA instead of DEXA due to its low cost and ease of application. Although our analyses consisted of 18 cancers, due to differences between cancers and small sample size of a certain cancer type, we failed to do the stratified analysis by cancer category, which may increase variability in data and decrease the statistical power. Furthermore, it was not possible to investigate a causal association between body composition phenotypes and all-cause mortality due to body composition analysis being detected just at baseline and without information on the change of body composition index.

## Conclusion

Our findings showed that a significant non-linear association between RFL and all-cause mortality, which were observed only in elderly men, might be attenuated after adjusting for CRP. Future work should be conducted to further explore these associations in high-quality longitudinal cohorts to deeply understand the mechanism of fat mass to lean body mass in cancer patients.

## Data Availability Statement

The raw data supporting the conclusions of this article will be made available by the authors, without undue reservation.

## Ethics Statement

The studies involving human participants were reviewed and approved by the INSCOC Research Ethics Committee and the Institutional Review Boards of all participating institutions (The First Hospital of Hebei Medical University, Tongji Hospital, The First Affiliated Hospital of Kunming Medical University, The First Hospital of Jilin University, Zhengzhou University, and Beijing Shijitan Hospital) and the study have been registered in Chinese Clinical Trial Register (URL: chictr.org.cn) (Register No: ChiCTR1800020329). The patients/participants provided their written informed consent to participate in this study.

## Author Contributions

ZL and HS: conceptualization and project administration. HXX and CS: methodology. SS: software. HD and BL: validation. HMX: formal analysis and writing (original draft preparation). YX and YZ: investigation. HQ, JC, KW, and WL: data curation. HMX: writing (original draft preparation). ZL, CS, HXX, and HS: supervision. ZL: funding acquisition. All authors have read and agreed to the published version of the manuscript.

## Conflict of Interest

The authors declare that the research was conducted in the absence of any commercial or financial relationships that could be construed as a potential conflict of interest.

## Publisher’s Note

All claims expressed in this article are solely those of the authors and do not necessarily represent those of their affiliated organizations, or those of the publisher, the editors and the reviewers. Any product that may be evaluated in this article, or claim that may be made by its manufacturer, is not guaranteed or endorsed by the publisher.

## Footnotes

1
http://www.chictr.org.cn/index.aspx

## References

[1] Ng M Fleming T Robinson M Thomson B Graetz N Margono C Global, regional, and national prevalence of overweight and obesity in children and adults during 1980-2013: a systematic analysis for the Global Burden of Disease Study 2013. *Lancet.* (2014) 384:766–81. 24880830 10.1016/S0140-6736(14)60460-8 PMC4624264

[2] Mu L Liu J Zhou G Wu C Chen B Lu Y Obesity prevalence and risks among Chinese adults: findings from the China PEACE million persons project, 2014-2018. *Circ Cardiovasc Qual Outcomes.* (2021) 14:e007292. 10.1161/CIRCOUTCOMES.120.007292 34107739 PMC8204767

[3] Simmonds M Llewellyn A Owen CG Woolacott N. Predicting adult obesity from childhood obesity: a systematic review and meta-analysis. *Obes Rev.* (2016) 17:95–107. 10.1111/obr.12334 26696565

[4] Adams KF Schatzkin A Harris TB Kipnis V Mouw T Ballard-Barbash R Overweight, obesity, and mortality in a large prospective cohort of persons 50 to 71 years old. *N Engl J Med.* (2006) 355:763–78. 10.1056/NEJMoa055643 16926275

[5] Berrington de Gonzalez A Hartge P Cerhan JR Flint AJ Hannan L MacInnis RJ Body-mass index and mortality among 1.46 million white adults. *N Engl J Med.* (2010) 363:2211–9. 10.1056/NEJMoa1000367 21121834 PMC3066051

[6] Chen Z Yang G Offer A Zhou M Smith M Peto R Body mass index and mortality in China: a 15-year prospective study of 220 000 men. *Int J Epidemiol.* (2012) 41:472–81. 10.1093/ije/dyr208 22296991

[7] Engin A. Obesity-associated breast cancer: analysis of risk factors. *Adv Exp Med Biol.* (2017) 960:571–606. 10.1007/978-3-319-48382-5_25 28585217

[8] Bardou M Barkun AN Martel M. Obesity and colorectal cancer. *Gut.* (2013) 62:933–47. 10.1136/gutjnl-2013-304701 23481261

[9] Manson JE Bassuk SS Hu FB Stampfer MJ Colditz GA Willett WC. Estimating the number of deaths due to obesity: can the divergent findings be reconciled? *J Womens Health.* (2007) 16:168–76. 10.1089/jwh.2006.0080 17388733

[10] Lee DH Keum N Hu FB Orav EJ Rimm EB Willett WC Predicted lean body mass, fat mass, and all cause and cause specific mortality in men: prospective US cohort study. *BMJ.* (2018) 362:k2575. 10.1136/bmj.k2575 29970408 PMC6028901

[11] Bandera EV Qin B Lin Y Zeinomar N Xu B Chanumolu D Association of body mass index, central obesity, and body composition with mortality among black breast cancer survivors. *JAMA Oncol.* (2021) 7:1–10. 10.1001/jamaoncol.2021.1499 34086040 PMC8377573

[12] van Dijk DP Bakens MJ Coolsen MM Rensen SS van Dam RM Bours MJ Low skeletal muscle radiation attenuation and visceral adiposity are associated with overall survival and surgical site infections in patients with pancreatic cancer. *J Cachexia Sarcopenia Muscle.* (2017) 8:317–26. 10.1002/jcsm.12155 27897432 PMC5377384

[13] Huang DD Wu GF Luo X Song HN Wang WB Liu NX Value of muscle quality, strength and gait speed in supporting the predictive power of GLIM-defined malnutrition for postoperative outcomes in overweight patients with gastric cancer. *Clin Nutr.* (2021) 40:4201–8. 10.1016/j.clnu.2021.01.038 33583658

[14] Caan BJ Cespedes Feliciano EM Prado CM Alexeeff S Kroenke CH Bradshaw P Association of muscle and adiposity measured by computed tomography with survival in patients with nonmetastatic breast cancer. *JAMA Oncol.* (2018) 4:798–804. 10.1001/jamaoncol.2018.0137 29621380 PMC6584322

[15] Baracos VE Mazurak VC Bhullar AS. Cancer cachexia is defined by an ongoing loss of skeletal muscle mass. *Ann Palliat Med.* (2019) 8:3–12. 10.21037/apm.2018.12.01 30685982

[16] Zhang X Li X Shi H Zhang K Zhang Q Tang M Association of the fat-free mass index with mortality in patients with cancer: a multicenter observational study. *Nutrition.* (2021) 94:111508. 10.1016/j.nut.2021.111508 34813982

[17] Oppert JM Charles MA Thibult N Guy-Grand B Eschwège E Ducimetière P. Anthropometric estimates of muscle and fat mass in relation to cardiac and cancer mortality in men: the paris prospective study. *Am J Clin Nutr.* (2002) 75:1107–13. 10.1093/ajcn/75.6.1107 12036820

[18] Willemsen ACH Degens J Baijens LWJ Dingemans AC Hoeben A Hoebers FJP Early loss of fat mass during chemoradiotherapy predicts overall survival in locally advanced squamous cell carcinoma of the lung, but not in locally advanced squamous cell carcinoma of the head and neck. *Front Nutr.* (2020) 7:600612. 10.3389/fnut.2020.600612 33324671 PMC7726186

[19] Cho Y Chang Y Ryu S Jung HS Kim CW Oh H Skeletal muscle mass to visceral fat area ratio as a predictor of NAFLD in lean and overweight men and women with effect modification by sex. *Hepatol Commun.* (2022) 3:1975. 10.1002/hep4.1975 35503803 PMC9426405

[20] Kim TN Park MS Lim KI Yang SJ Yoo HJ Kang HJ Skeletal muscle mass to visceral fat area ratio is associated with metabolic syndrome and arterial stiffness: the Korean Sarcopenic Obesity Study (KSOS). *Diabetes Res Clin Pract.* (2011) 93:285–91. 10.1016/j.diabres.2011.06.013 21752483

[21] Wang Q Zheng D Liu J Fang L Li Q. Skeletal muscle mass to visceral fat area ratio is an important determinant associated with type 2 diabetes and metabolic syndrome. *Diab Metab Syndr Obes.* (2019) 12:1399–407. 10.2147/DMSO.S211529 31616170 PMC6698596

[22] Draghia-Akli R Khan AS. Muscle and fat mass modulation in different clinical models. *Methods Mol Biol.* (2008) 423:449–60. 10.1007/978-1-59745-194-9_35 18370221

[23] Xu H Song C Wang C Fu Z Guo Z Lin Y Investigation on nutrition status and clinical outcome of patients with common cancers in Chinese patients: a multicenter prospective study protocol. *Int J Clin Trial.* (2020) 7:1–9. 10.18203/2349-3259.ijct20201052

[24] Kondrup J Rasmussen HH Hamberg O Stanga Z. Nutritional risk screening (NRS 2002): a new method based on an analysis of controlled clinical trials. *Clin Nutr.* (2003) 22:321–36. 10.1016/s0261-5614(02)00214-5 12765673

[25] Koom WS Ahn SD Song SY Lee CG Moon SH Chie EK Nutritional status of patients treated with radiotherapy as determined by subjective global assessment. *Radiat Oncol J.* (2012) 30:132–9. 10.3857/roj.2012.30.3.132 23170292 PMC3496847

[26] Terret C Albrand G Moncenix G Droz JP. Karnofsky performance scale (KPS) or physical performance test (PPT)? THAT is the question. *Crit Rev Oncol Hematol.* (2011) 77:142–7. 10.1016/j.critrevonc.2010.01.015 20185330

[27] Aaronson NK Ahmedzai S Bergman B Bullinger M Cull A Duez NJ The European organization for research and treatment of cancer QLQ-C30: a quality-of-life instrument for use in international clinical trials in oncology. *J Natl Cancer Inst.* (1993) 85:365–76. 10.1093/jnci/85.5.365 8433390

[28] Kernan WN Viscoli CM Brass LM Broderick JP Brott T Feldmann E Phenylpropanolamine and the risk of hemorrhagic stroke. *N Engl J Med.* (2000) 343:1826–32. 11117973 10.1056/NEJM200012213432501

[29] Anyżewska A ł́akomy R Lepionka T Szarska E Maculewicz E Tomczak A Association between diet, physical activity and body mass index, fat mass index and bone mineral density of soldiers of the Polish air cavalry units. *Nutrients.* (2020) 12:242. 10.3390/nu12010242 31963454 PMC7019523

[30] Young HJ Southern WM McCully KK. Comparisons of ultrasound-estimated intramuscular fat with fitness and health indicators. *Muscle Nerve.* (2016) 54:743–9. 10.1002/mus.25105 26969901

[31] Goossens GH. The metabolic phenotype in obesity: fat mass, body fat distribution, and adipose tissue function. *Obes Facts.* (2017) 10:207–15. 10.1159/000471488 28564650 PMC5644968

[32] Kolb R Sutterwala FS Zhang W. Obesity and cancer: inflammation bridges the two. *Curr Opin Pharmacol.* (2016) 29:77–89. 10.1016/j.coph.2016.07.005 27429211 PMC4992602

[33] Black S Kushner I Samols D. C-reactive Protein. *J Biol Chem.* (2004) 279:48487–90. 10.1074/jbc.R400025200 15337754

[34] Pathak A Agrawal A. Evolution of C-reactive protein. *Front Immunol.* (2019) 10:943. 10.3389/fimmu.2019.00943 31114584 PMC6503050

[35] Deng T Lyon CJ Bergin S Caligiuri MA Hsueh WA. Obesity, inflammation, and cancer. *Annu Rev Pathol.* (2016) 11:421–49. 10.1146/annurev-pathol-012615-044359 27193454

[36] Iyengar NM Gucalp A Dannenberg AJ Hudis CA. Obesity and cancer mechanisms: tumor microenvironment and inflammation. *J Clin Oncol.* (2016) 34:4270–6. 10.1200/JCO.2016.67.4283 27903155 PMC5562428

[37] Jiralerspong S Goodwin PJ. Obesity and breast cancer prognosis: evidence, challenges, and opportunities. *J Clin Oncol.* (2016) 34:4203–16. 10.1200/JCO.2016.68.4480 27903149

[38] Wiegert EVM de Oliveira LC Calixto-Lima L Borges NA Rodrigues J da Mota ESLMS Association between low muscle mass and survival in incurable cancer patients: a systematic review. *Nutrition.* (2020) 72:110695. 10.1016/j.nut.2019.110695 32007806

[39] Fielding RA Vellas B Evans WJ Bhasin S Morley JE Newman AB Sarcopenia: an undiagnosed condition in older adults. Current consensus definition: prevalence, etiology, and consequences. International working group on sarcopenia. *J Am Med Dir Assoc.* (2011) 12:249–56. 10.1016/j.jamda.2011.01.003 21527165 PMC3377163

[40] Hori Y Hoshino M Inage K Miyagi M Takahashi S Ohyama S ISSLS PRIZE IN CLINICAL SCIENCE 2019: clinical importance of trunk muscle mass for low back pain, spinal balance, and quality of life-a multicenter cross-sectional study. *Eur Spine J.* (2019) 28:914–21. 10.1007/s00586-019-05904-7 30729293

[41] Balogun S Winzenberg T Wills K Scott D Jones G Callisaya ML Prospective associations of low muscle mass and strength with health-related quality of life over 10-year in community-dwelling older adults. *Exp Gerontol.* (2019) 118:65–71. 10.1016/j.exger.2019.01.008 30641106

