# Supplementary material for: Association Between Fat Mass to Lean Body Mass Ratio and All-Cause Mortality Among Middle-Aged and Elderly Cancer Patients Without Obesity: A Multi-Center Observational Study in China
Source: Front Nutr. 2022 Jun 16;9:914020. doi: 10.3389/fnut.2022.914020 (PMC9249599; doi:10.3389/fnut.2022.914020)
Supplement: Supplementary file 1 [file Data_Sheet_1.docx]

Supplementary Material


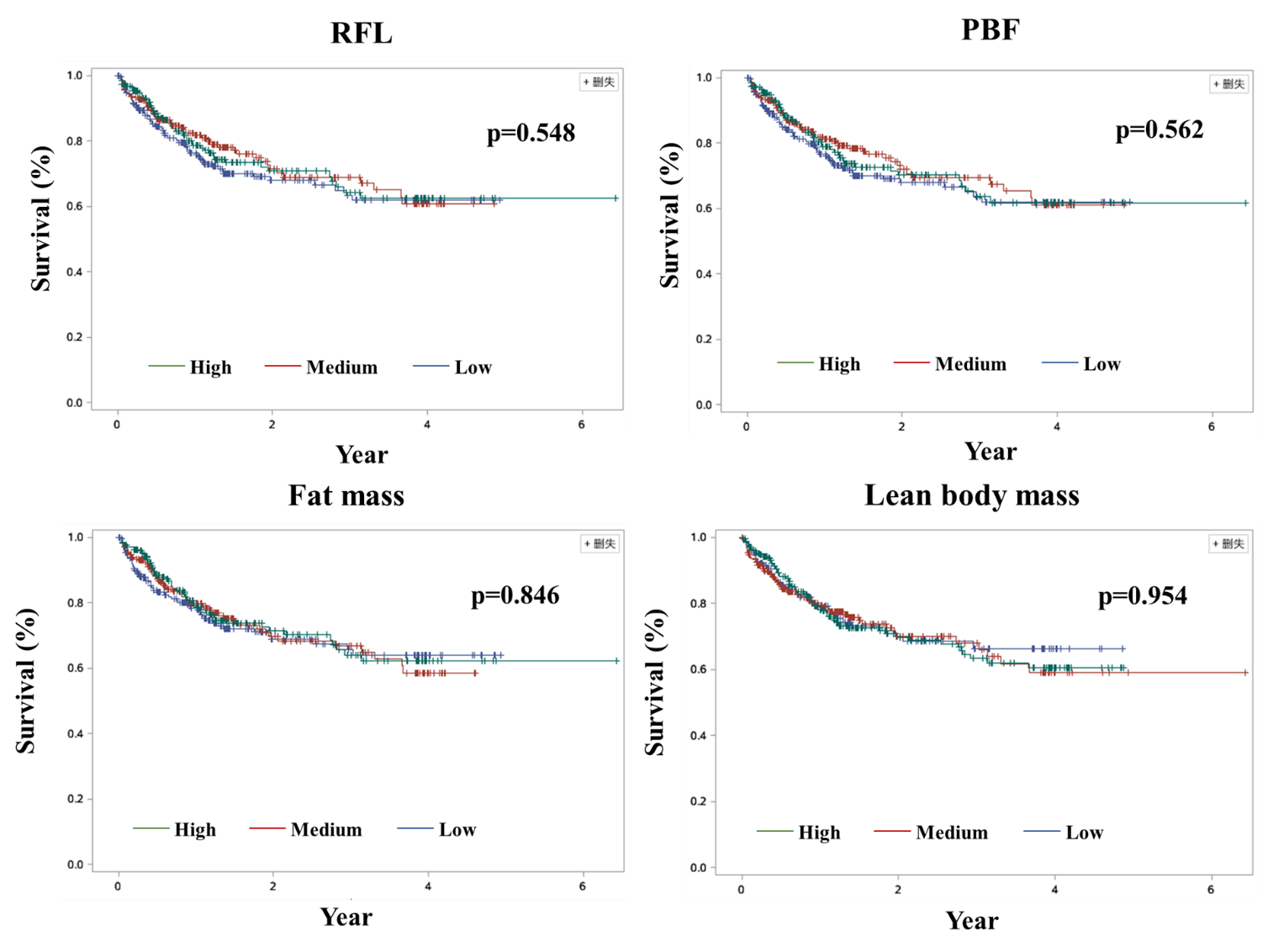


**Supplementary Figure 1.** Kaplane-Meier curves among men cancer patients aged 40-60 years, Fat mass to lean body mass ratio; PBF: Percentage of body fat


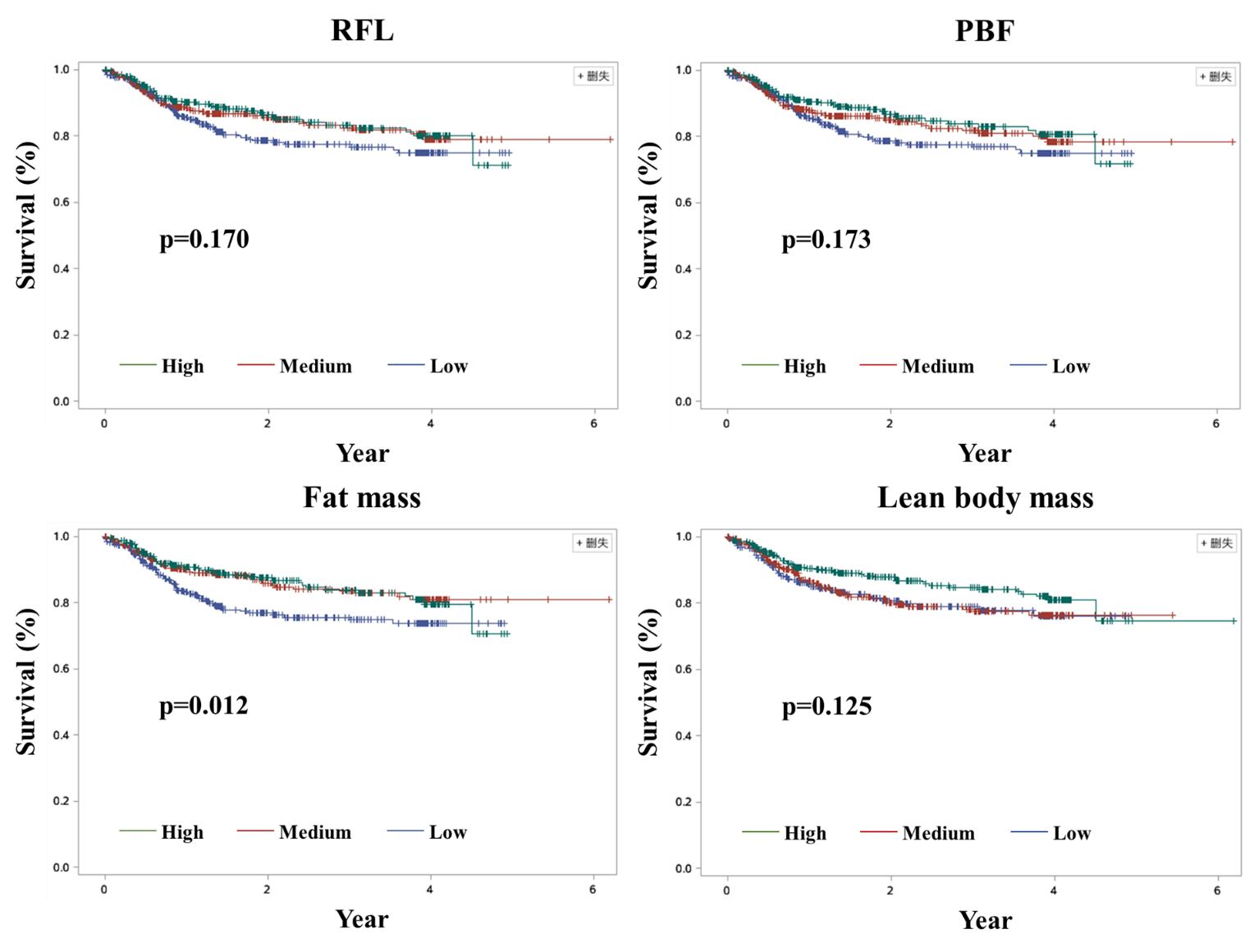


**Supplementary Figure 2.** Kaplane-Meier curves among women cancer patients aged 40-60 years, Fat mass to lean body mass ratio; PBF: Percentage of body fat.


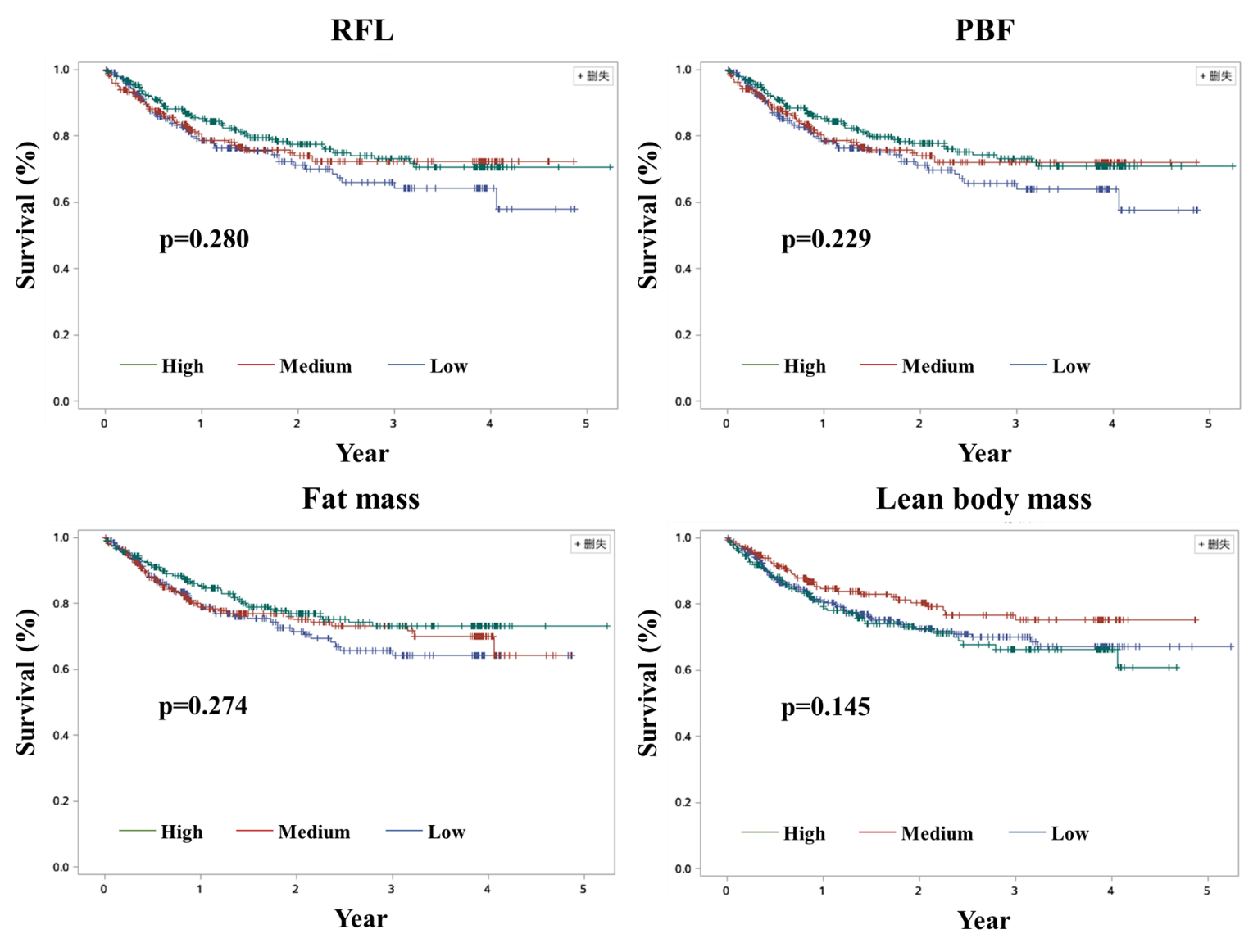


**Supplementary Figure 3.** Kaplane-Meier curves among women cancer patients aged ≥ 60 years, Fat mass to lean body mass ratio; PBF: Percentage of body fat.


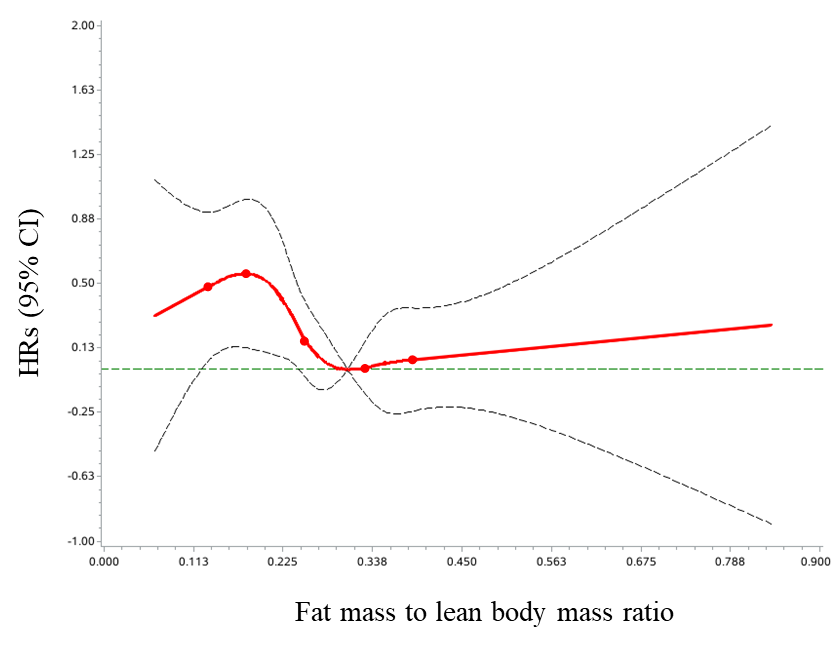


**Supplementary Figure 4.** Restricted cubic spline among men cancer patients aged ≥ 60 years. Data shown in the figure were adjusted for age, education, smoking, alcohol, family history of cancer, version of Body Composition Analyzer, Karnofsky performance scores, duration of hospital stays, NRS 2002 score, nutrition support, handgrip strength, commodities, previous treatments, cancer types, TNM stages, and quality of life;

**Supplementary Table 1.** Association between lean body mass and all-cause mortality using Cox proportional hazard regression

|  | **<60 years** | | | p | ≥60 years | | | p |
| --- | --- | --- | --- | --- | --- | --- | --- | --- |
|  | **T1** | **T2** | **T3** |  | **T1** | **T2** | **T3** |  |
| **Female** |  |  |  |  |  |  |  |  |
| Unadjusted model | 1 | 0.97 (0.66, 1.43) | 0.70 (0.47, 1.04) | 0.128 | 1 | 0.72 (0.49, 1.07) | 1.07 (0.75, 1.54) | 0.148 |
| Model A^b^ | 1 | 0.99 (0.67, 1.45) | 0.70 (0.47, 1.04) | 0.118 | 1 | 0.74 (0.49, 1.10) | 1.14 (0.79, 1.67) | 0.124 |
| Model B^b^ | 1 | 1.10 (0.75, 1.64) | 0.80 (0.53, 1.21) | 0.241 | 1 | 0.77 (0.52, 1.16) | 1.30 (0.89, 1.91) | 0.068 |
| Model C^b^ | 1 | 0.80 (0.51, 1.31) | 0.88 (0.54, 1.43) | 0.696 | 1 | 0.82 (0.53, 1.28) | 1.46 (0.94, 2.28) | 0.047 |
| Model D^b^ | 1 | 1.20 (0.62, 2.33) | 1.44 (0.74, 2.82) | 0.557 | 1 | 0.83 (0.43, 1.61) | 2.07 (1.11, 3.88) | 0.024 |
| **Male** |  |  |  |  |  |  |  |  |
| Unadjusted model | 1 | 1.06 (0.71, 1.57) | 1.05 (0.72, 1.55) | 0.954 | 1 | 0.79 (0.59, 1.07) | 0.89 (0.66, 1.19) | 0.304 |
| Model A^b^ | 1 | 1.03 (0.69, 1.54) | 1.02 (0.69, 1.51) | 0.988 | 1 | 0.83 (0.61, 1.13) | 0.93 (0.69, 1.27) | 0.497 |
| Model B^b^ | 1 | 1.14 (0.75, 1.72) | 1.17 (0.76, 1.80) | 0.760 | 1 | 0.90 (0.66, 1.23) | 0.97 (0.70, 1.33) | 0.812 |
| Model C^b^ | 1 | 1.11 (0.66, 1.87) | 1.62 (0.94, 2.78) | 0.139 | 1 | 1.03 (0.73, 1.46) | 0.88 (0.61, 1.26) | 0.680 |
| Model D^b^ | 1 | 0.89 (0.49, 1.65) | 1.14 (0.60, 2.16) | 0.699 | 1 | 0.95 (0.60, 1.48) | 0.83 (0.52, 1.31) | 0.713 |

^a^ Values are models adjusted HR and 95% confidence interval, ranges for tertiles (T) 1 through 3. Linear trends (p for trend) were obtained with lean body mass as continuous variables.

^b^ Model A: adjusted for age, education, smoking, alcohol, family history of cancer, version of Body Composition Analyzer; Model B: as model A and additionally adjusted for Karnofsky performance scores, duration of hospital stays, NRS 2002 score, nutrition support and handgrip strength; Model C: as Model B and additionally adjusted for commodities, previous treatments, cancer types TNM stages, and quality of life; Model D: as Model C and additionally adjusted C-reaction protein.

**Supplementary Table 2. The results of the effect value and p-value of variables in the univariate models**

| **Variables** | **Group** | **HRs** | **P value** | **Variables** | **Group** | **HRs** | **P value** |
| --- | --- | --- | --- | --- | --- | --- | --- |
| Gender | Male | 1 | <0.001 | **TNM stages** | Ⅰ | 1 | <0.001 |
|  | Female | 0.55 (0.47, 0.63) |  |  | Ⅱ | 0.82 (0.63, 1.07) |  |
| Age | <60 | 1 | <0.001 |  | Ⅲ | 0.61 (0.48, 0.79) |  |
|  | ≥60 | 1.51 (1.31, 1.75) |  |  | Ⅳ | 0.59 (0.45, 0.77) |  |
| Educate | Low | 1 | <0.001 | **Treatments** | Surgery | 1.15 (0.71, 1.84) | <0.001 |
|  | High | 0.73 (0.63, 0.86) |  |  | Chemotherapy | 0.65 (0.40, 1.05) |  |
| Smoke | None | 1 | <0.001 |  | Radiotherapy | 1.22 (0.77, 1.93) |  |
|  | Smoke ever | 1.56 (1.32, 1.84) |  |  | Others | 1 |  |
|  | Current smoking | 1.88 (1.56, 2.27) |  | RFL |  | 0.22 (0.14, 0.33) | <0.001 |
| Alcohol | No | 1 | 0.001 | Percentage of body fat | | 0.97 (0.96, 0.98) | <0.001 |
|  | Yes | 1.35 (1.13, 1.61) |  | Fat mass |  | 0.97 (0.96, 0.98) | <0.001 |
| Tea | No | 1 | 0.135 | Lean body mass | | 1.02 (1.01, 1.03) | <0.001 |
|  | Yes | 1.15 (0.96, 1.38) |  | Body mass index | | 0.95 (0.93, 0.97) | <0.001 |
| Family history of cancer | No | 1 | 0.047 | Handgrip strength | | 1.01 (1.00, 1.02) | 0.079 |
|  | Yes | 0.82 (0.67, 1.00) |  | Duration of hospital stays (days) | | 1.00 (0.99, 1.01) | 0.581 |
| Comorbidity | No | 1 | <0.0001 | NRS 2002 | <3 | 1 | <0.001 |
|  | Yes | 1.67 (1.43, 1.94) |  |  | ≥3 | 1.82 (1.51, 2.18) |  |
| KPS score |  | 0.98 (0.98, 0.99) | <0.001 | Nutrition support | No | 1 | <0.001 |
| Quality of life |  | 1.03 (1.02, 1.03) | <0.001 |  | Yes | 1.45 (1.19, 1.78) |  |
| C-reaction protein | | 1.00 (1.00, 1.01) | 0.002 | PG‐SGA | 0-1 | 1 | <0.001 |
|  |  |  |  |  | 2-8 | 1.45 (1.24, 1.69) |  |
|  |  |  |  |  | ≥9 | 2.80 (2.24, 3.49) |  |

NOTE: PG-SGA, patient-generated subjective nutrition assessment; NRS 2002, nutrition risk screening 2002; KPS, karnofsky performance scores; RFL: Ratio of fat mass to lean body mass.

**Supplementary Table 3. Association between RFL and all-cause mortality using the Cox regression model with each of covariables.**

|  | **HRs (95% CI)** | | | p |
| --- | --- | --- | --- | --- |
|  | **T1** | **T2** | **T3** |  |
| **Unadjusted model** | 1 | 0.76 (0.63, 0.90) | 0.78 (0.65, 0.92) | 0.002 |
| Gender | 1 | 0.75 (0.63, 0.89) | 0.78 (0.65, 0.92) | 0.001 |
| Age | 1 | 0.73 (0.62, 0.88) | 0.72 (0.60, 0.85) | 0.000 |
| Education level | 1 | 0.76 (0.64, 0.91) | 0.78 (0.66, 0.93) | 0.003 |
| Smoking | 1 | 0.77 (0.65, 0.92) | 0.80 (0.67, 0.95) | 0.006 |
| Alcohol | 1 | 0.76 (0.63, 0.90) | 0.78 (0.66, 0.93) | 0.003 |
| Tea | 1 | 0.75 (0.63, 0.90) | 0.77 (0.65, 0.92) | 0.002 |
| Family history of cancer | 1 | 0.76 (0.64, 0.91) | 0.78 (0.66, 0.93) | 0.001 |
| Commodities | 1 | 0.78 (0.65, 0.93) | 0.82 (0.69, 0.98) | 0.010 |
| Body mass index | 1 | 0.88 (0.73, 1.07) | 1.06 (0.84, 1.33) | 0.135 |
| Lean body mass | 1 | 0.75 (0.63, 0.89) | 0.77 (0.65, 0.92) | 0.001 |
| NRS 2002 score | 1 | 0.81 (0.68, 0.96) | 0.85 (0.71, 1.00) | 0.038 |
| PG-SGA | 1 | 0.81 (0.68, 0.96) | 0.85 (0.71, 1.01) | 0.038 |
| Nutrition support | 1 | 0.75 (0.63, 0.89) | 0.77 (0.64, 0.91) | 0.001 |
| Handgrip strength | 1 | 0.75 (0.63, 0.89) | 0.77 (0.65, 0.92) | 0.001 |
| Duration of hospital stays (days) | 1 | 0.75 (0.63, 0.90) | 0.77 (0.65, 0.92) | 0.002 |
| KPS | 1 | 0.77 (0.64, 0.91) | 0.79 (0.66, 0.94) | 0.004 |
| Treatments | 1 | 0.74 (0.62, 0.88) | 0.76 (0.64, 0.91) | 0.001 |
| Cancer types | 1 | 0.80 (0.6, 0.96) | 0.86 (0.72, 1.02) | 0.039 |
| TNM stage | 1 | 0.78 (0.64, 0.95) | 0.80 (0.65, 0.97) | 0.019 |
| Quality of life | 1 | 0.79 (0.66, 0.94) | 0.79 (0.67, 0.94) | 0.009 |
| C-reaction protein | 1 | 0.83 (0.66, 1.03) | 0.77 (0.61, 0.96) | 0.056 |

NOTE: PG-SGA, patient-generated subjective nutrition assessment; NRS 2002, nutrition risk screening 2002; KPS, karnofsky performance scores; RFL: Ratio of fat mass to lean body mass.

*P value were association between RFL and all-cause mortality using the Cox regression model when each of above and RFL were put into the model together.

**Supplementary Table 4.** Association between fat mass to lean body mass ratio and all-cause mortality using Cox proportional hazard regression (medium as reference) ^a^

|  | **<60 years** | | | p | ≥60 years | | | P for trend |
| --- | --- | --- | --- | --- | --- | --- | --- | --- |
|  | **T1** | **T2** | **T3** |  | **T1** | **T2** | **T3** |  |
| **Female** |  |  |  |  |  |  |  |  |
| Unadjusted model | 1.31 (0.90, 1.89) | 1 | 0.94 (0.62, 1.42) | 0.172 | 1.19 (0.81, 1.76) | 1 | 0.88 (0.60, 1.28) | 0.282 |
| Model A^b^ | 1.27 (0.87, 1.84) | 1 | 0.87 (0.58, 1.33) | 0.159 | 1.05 (0.71, 1.57) | 1 | 0.88 (0.60, 1.28) | 0.624 |
| Model B^b^ | 1.01 (0.69, 1.49) | 1 | 0.85 (0.56, 1.29) | 0.661 | 0.94 (0.62, 1.41) | 1 | 0.90 (0.61, 1.32) | 0.857 |
| Model C^b^ | 0.96 (0.61, 1.52) | 1 | 0.91 (0.54, 1.52) | 0.935 | 0.77 (0.48, 1.22) | 1 | 0.85 (0.55, 1.31) | 0.521 |
| Model D^b^ | 1.16 (0.65, 2.07) | 1 | 0.77 (0.37, 1.59) | 0.486 | 0.48 (0.25, 0.93) | 1 | 0.43 (0.23, 0.82) | 0.018 |
| **Male** |  |  |  |  |  |  |  |  |
| Unadjusted model | 1.20 (0.85, 1.71) | 1 | 1.04 (0.71, 1.53) | 0.549 | 1.74 (1.27, 2.37) | 1 | 1.15 (0.84, 1.57) | 0.001 |
| Model A^b^ | 1.19 (0.83, 1.69) | 1 | 1.05 (0.71, 1.55) | 0.610 | 1.72 (1.26, 2.36) | 1 | 1.08 (0.78, 1.48) | 0.001 |
| Model B^b^ | 1.16 (0.81, 1.67) | 1 | 1.14 (0.77, 1.69) | 0.682 | 1.74 (1.27, 2.40) | 1 | 1.08 (0.78, 1.48) | 0.001 |
| Model C^b^ | 1.25 (0.79, 1.97) | 1 | 1.25 (0.78, 2.02) | 0.569 | 1.53 (1.06, 2.21) | 1 | 1.01 (0.71, 1.44) | 0.033 |
| Model D^b^ | 1.02 (0.56, 1.85) | 1 | 1.27 (0.70, 2.29) | 0.653 | 1.34 (0.84, 2.16) | 1 | 1.13 (0.73, 1.75) | 0.475 |

^a^ Values are models adjusted HR and 95% confidence interval, ranges for tertiles (T) 1 through 3. Linear trends (p for trend) were obtained with ratio of fat mass to fat-free mass as continuous variables.

^b^ Model A: adjusted for age, education, smoking, alcohol, family history of cancer, version of Body Composition Analyzer; Model B: as model A and additionally adjusted for Karnofsky performance scores, duration of hospital stays, NRS 2002 score, nutrition support and handgrip strength; Model C: as Model B and additionally adjusted for commodities, previous treatments, cancer types TNM stages, and quality of life; Model D: as Model C and additionally adjusted C-reaction protein.

**Supplementary Table 5.** Correlation coefficients between body composition indicators.

|  | BMI | FM | PBF | LM | RFL |
| --- | --- | --- | --- | --- | --- |
| BMI | 1.000 |  |  |  |  |
| FM | 0.850 | 1.000 |  |  |  |
| PBF | 0.645 | 0.911 | 1.000 |  |  |
| LM | 0.303 | -0.028 | -0.392 | 1.000 |  |
| RFL | 0.649 | 0.904 | 0.980 | -0.418 | 1.000 |

BMI: Body mass index; FM: Fat mass; PBF: Percentage of body fat; LM: Lean body mass; RFL: fat mass to lean body mass ratio

**Supplementary Table 6.** Sensitive analysis for fat mass and all-cause mortality.

|  | **<60 years** | | | p | ≥60 years | | | p |
| --- | --- | --- | --- | --- | --- | --- | --- | --- |
|  | **T1** | **T2** | **T3** |  | **T1** | **T2** | **T3** |  |
| **Female** |  |  |  |  |  |  |  |  |
| Model C | 1 | 0.90 (0.56, 1.44) | 0.89 (0.55, 1.45) | 0.866 | 1 | 1.28 (0.79, 2.07) | 1.27 (0.79, 2.02) | 0.528 |
| Model C + BMI | 1 | 1.04 (0.60, 1.78) | 1.22 (0.56, 2.67) | 0.859 | 1 | 1.15 (0.67, 1.99) | 1.07 (0.51, 2.26) | 0.853 |
| Model C + BMI+ CRP | 1 | 0.86 (0.41, 1.78) | 0.64 (0.23, 1.82) | 0.705 | 1 | 1.40 (0.66, 2.97) | 0.61 (0.21, 1.79) | 0.059 |
| Model C + LBM | 1 | 0.91 (0.57, 1.45) | 0.93 (0.56, 1.52) | 0.910 | 1 | 1.21 (0.76, 1.92) | 1.16 (0.73, 1.84) | 0.709 |
| Model C + LBM + CRP | 1 | 0.93 (0.49, 1.75) | 0.73 (0.39, 1.39) | 0.636 | 1 | 1.81 (0.97, 3.40) | 0.98 (0.50, 1.96) | 0.077 |
| **Male** |  |  |  |  |  |  |  |  |
| Model C | 1 | 1.13 (0.71, 1.80) | 1.14 (0.72, 1.81) | 0.824 | 1 | 0.71 (0.49, 1.01) | 0.70 (0.49, 1.02) | 0.097 |
| Model C + BMI | 1 | 1.02 (0.62, 1.69) | 0.88 (0.45, 1.70) | 0.862 | 1 | 0.75 (0.50, 1.13) | 0.80 (0.47, 1.36) | 0.387 |
| Model C + BMI+ CRP | 1 | 1.10 (0.58, 2.10) | 0.99 (0.45, 2.16) | 0.927 | 1 | 0.96 (0.57, 1.61) | 0.92 (0.47, 1.80) | 0.972 |
| Model C + LBM | 1 | 1.10 (0.69, 1.75) | 1.05 (0.65, 1.68) | 0.929 | 1 | 0.69 (0.48, 0.99) | 0.67 (0.45, 0.98) | 0.064 |
| Model C + LBM + CRP | 1 | 1.26 (0.68, 2.32) | 1.37 (0.76, 2.49) | 0.566 | 1 | 0.91 (0.57, 1.44) | 0.83 (0.51, 1.35) | 0.747 |

BMI: Body mass index; FM: Fat mass; CRP: C-reaction protein

**Model C:** adjusted for age, education, smoking, alcohol, family history of cancer, version of Body Composition Analyzer, Karnofsky performance scores, duration of hospital stays, NRS 2002 score, nutrition support and handgrip strength, commodities, previous treatments, cancer types TNM stages, and quality of life.

**Supplementary Table 7.** Sensitive analysis for lean body mass and all-cause mortality.

|  | **<60 years** | | | p | ≥60 years | | | p |
| --- | --- | --- | --- | --- | --- | --- | --- | --- |
|  | **T1** | **T2** | **T3** |  | **T1** | **T2** | **T3** |  |
| **Female** |  |  |  |  |  |  |  |  |
| Model C | 1 | 0.80 (0.51, 1.31) | 0.88 (0.54, 1.43) | 0.696 | 1 | 0.82 (0.53, 1.28) | 1.46 (0.94, 2.28) | 0.047 |
| Model C + BMI | 1 | 0.86 (0.53, 1.38) | 1.00 (0.58, 1.72) | 0.759 | 1 | 0.83 (0.52, 1.31) | 1.46 (0.90, 2.36) | 0.071 |
| Model C + BMI+ CRP | 1 | 1.14 (0.59, 2.18) | 1.67 (0.88, 3.15) | 0.374 | 1 | 0.71 (0.37, 1.40) | 1.66 (0.84, 2.27) | 0.066 |
| Model C + FM | 1 | 0.84 (0.52, 1.35) | 0.95 (0.57, 1.56) | 0.751 | 1 | 0.83 (0.53, 1.30) | 1.49 (0.95, 2.33) | 0.054 |
| Model C + FM + CRP | 1 | 1.12 (0.59, 2.14) | 1.52 (0.77, 3.00) | 0.403 | 1 | 0.73 (0.38, 1.40) | 1.72 (0.92, 2.41) | 0.049 |
| Model C + PBF | 1 | 0.82 (0.51, 1.32) | 0.88 (0.54, 1.43) | 0.712 | 1 | 0.84 (0.54, 1.31) | 1.53 (0.99, 2.36) | 0.042 |
| Model C + PBF + CRP | 1 | 1.08 (0.56, 2.06) | 1.38 (0.71, 2.69) | 0.561 | 1 | 0.73 (0.38, 1.39) | 1.76 (0.97, 3.20) | 0.038 |
| **Male** |  |  |  |  |  |  |  |  |
| Model C | 1 | 1.11 (0.66, 1.87) | 1.62 (0.94, 2.78) | 0.139 | 1 | 1.03 (0.73, 1.46) | 0.88 (0.61, 1.26) | 0.680 |
| Model C + BMI | 1 | 1.12 (0.66, 1.90) | 1.58 (0.85, 2.95) | 0.263 | 1 | 1.06 (0.76, 1.46) | 1.30 (0.89, 1.90) | 0.360 |
| Model C + BMI+ CRP | 1 | 0.89 (0.46, 1.74) | 0.95 (0.45, 2.01) | 0.935 | 1 | 1.02 (0.64, 1.63) | 0.84 (0.49, 1.44) | 0.739 |
| Model C + FM | 1 | 1.13 (0.68, 1.89) | 1.63 (0.94, 2.83) | 0.149 | 1 | 1.12 (0.79, 1.58) | 0.95 (0.65, 1.39) | 0.696 |
| Model C + FM + CRP | 1 | 1.00 (0.52, 1.92) | 1.20 (0.61, 2.34) | 0.802 | 1 | 0.99 (0.64, 1.56) | 0.80 (0.50, 1.29) | 0.608 |
| Model C + PBF | 1 | 1.13 (0.67, 1.89) | 1.62 (0.95, 2.78) | 0.139 | 1 | 1.08 (0.76, 1.52) | 0.88 (0.61, 1.27) | 0.585 |
| Model C + PBF + CRP | 1 | 1.04 (0.54, 1.98) | 1.28 (0.66, 2.48) | 0.681 | 1 | 0.99 (0.64, 1.54) | 0.79 (0.50, 1.25) | 0.561 |

BMI: Body mass index; FM: Fat mass; CRP: C-reaction protein;

**Model C:** adjusted for age, education, smoking, alcohol, family history of cancer, version of Body Composition Analyzer, Karnofsky performance scores, duration of hospital stays, NRS 2002 score, nutrition support and handgrip strength, commodities, previous treatments, cancer types TNM stages, and quality of life.

**Supplementary Table 8.** Sensitive analysis for fat mass to lean body mass ratio and all-cause mortality.

|  | **<60 years** | | | p | ≥60 years | | | p |
| --- | --- | --- | --- | --- | --- | --- | --- | --- |
|  | **T1** | **T2** | **T3** |  | **T1** | **T2** | **T3** |  |
| **Female** |  |  |  |  |  |  |  |  |
| Model C | 1 | 1.04 (0.66, 1.64) | 0.95 (0.57, 1.56) | 0.935 | 1 | 0.84 (0.57, 1.24) | 0.74 (0.51, 1.07) | 0.282 |
| Model C + BMI | 1 | 1.11 (0.73, 1.71) | 1.33 (0.76, 2.30) | 0.605 | 1 | 1.08 (0.68, 1.71) | 0.98 (0.56, 1.71) | 0.873 |
| Model C + BMI+ CRP | 1 | 0.96 (0.55, 1.67) | 0.81 (0.36, 1.78) | 0.857 | 1 | 1.59 (0.84, 3.04) | 1.00 (0.45, 2.25) | 0.160 |
| Model C + LBM | 1 | 1.02 (0.69, 1.51) | 1.11 (0.73, 1.69) | 0.870 | 1 | 1.16 (0.77, 1.77) | 1.11 (0.74, 1.68) | 0.776 |
| Model C + LBM + CRP | 1 | 0.97 (0.58, 1.61) | 0.82 (0.45, 1.49) | 0.800 | 1 | 1.69 (0.94, 3.03) | 1.12 (0.60, 2.08) | 0.150 |
| **Male** |  |  |  |  |  |  |  |  |
| Model C | 1 | 0.80 (0.51, 1.27) | 1.00 (0.64, 1.57) | 0.569 | 1 | 0.65 (0.45, 0.94) | 0.66 (0.46, 0.95) | 0.033 |
| Model C + BMI | 1 | 0.78 (0.52, 1.16) | 0.74 (0.44, 1.25) | 0.396 | 1 | 0.60 (0.42, 0.85) | 0.68 (0.45, 1.03) | 0.017 |
| Model C + BMI+ CRP | 1 | 0.99 (0.60, 1.63) | 0.87 (0.46, 1.66) | 0.890 | 1 | 0.67 (0.43, 1.03) | 0.82 (0.49, 1.38) | 0.172 |
| Model C + LBM | 1 | 0.85 (0.59, 1.24) | 0.93 (0.63, 1.36) | 0.703 | 1 | 0.57 (0.41, 0.79) | 0.63 (0.46, 0.86) | 0.001 |
| Model C + LBM + CRP | 1 | 1.11 (0.69, 1.79) | 1.20 (0.74, 1.96) | 0.757 | 1 | 0.64 (0.42, 0.96) | 0.75 (0.50, 1.10) | 0.093 |

BMI: Body mass index; FM: Fat mass; CRP: C-reaction protein

**Model C:** adjusted for age, education, smoking, alcohol, family history of cancer, version of Body Composition Analyzer, Karnofsky performance scores, duration of hospital stays, NRS 2002 score, nutrition support and handgrip strength, commodities, previous treatments, cancer types TNM stages, and quality of life.

**Supplementary Table 9.** Sensitive analysis for percentage of body fat and all-cause mortality.

|  | **<60 years** | | | p | ≥60 years | | | p |
| --- | --- | --- | --- | --- | --- | --- | --- | --- |
|  | **T1** | **T2** | **T3** |  | **T1** | **T2** | **T3** |  |
| **Female** |  |  |  |  |  |  |  |  |
| Model C | 1 | 1.07 (0.68, 1.69) | 0.88 (0.53, 1.46) | 0.746 | 1 | 1.22 (0.77, 1.93) | 1.07 (0.67, 1.70) | 0.695 |
| Model C + BMI | 1 | 1.14 (0.74, 1.73) | 1.24 (0.71, 2.16) | 0.735 | 1 | 1.06 (0.67, 1.68) | 0.97 (0.55, 1.69) | 0.898 |
| Model C + BMI+ CRP | 1 | 0.99 (0.57, 1.71) | 0.75 (0.33, 1.67) | 0.709 | 1 | 1.44 (0.76, 2.73) | 0.90 (0.41, 1.99) | 0.213 |
| Model C + LBM | 1 | 1.06 (0.72, 1.56) | 1.07 (0.70, 1.63) | 0.940 | 1 | 1.14 (0.75, 1.72) | 1.10 (0.73, 1.67) | 0.818 |
| Model C + LBM + CRP | 1 | 1.02 (0.62, 1.67) | 0.78 (0.43, 1.43) | 0.671 | 1 | 1.56 (0.88, 2.79) | 1.05 (0.57, 1.93) | 0.219 |
| **Male** |  |  |  |  |  |  |  |  |
| Model C | 1 | 0.75 (0.48, 1.18) | 0.96 (0.61, 1.50) | 0.431 | 1 | 0.70 (0.49, 1.01) | 0.70 (0.49, 1.00) | 0.082 |
| Model C + BMI | 1 | 0.75 (0.50, 1.12) | 0.70 (0.42, 1.19) | 0.306 | 1 | 0.63 (0.44, 0.88) | 0.73 (0.48, 1.09) | 0.028 |
| Model C + BMI+ CRP | 1 | 0.95 (0.58, 1.56) | 0.85 (0.45, 1.63) | 0.886 | 1 | 0.69 (0.45, 1.06) | 0.90 (0.54, 1.51) | 0.179 |
| Model C + LBM | 1 | 0.83 (0.57, 1.20) | 0.90 (0.61, 1.32) | 0.611 | 1 | 0.59 (0.43, 0.82) | 0.64 (0.47, 0.88) | 0.003 |
| Model C + LBM + CRP | 1 | 1.07 (0.67, 1.72) | 1.18 (0.72, 1.94) | 0.797 | 1 | 0.65 (0.43, 0.98) | 0.78 (0.52, 1.15) | 0.121 |

BMI: Body mass index; FM: Fat mass; CRP: C-reaction protein

**Model C:** adjusted for age, education, smoking, family history of cancer, version of Body Composition Analyzer, Karnofsky performance scores, NRS 2002 score, nutrition support and handgrip strength, commodities, previous treatments, cancer types TNM stages, and quality of life.

**Supplementary Table 10.** Association between fat mass to lean body mass ratio and all-cause mortality among patients with BMI >30 kg/m^2^ using Cox proportional hazard regression ^a^

|  | **<60 years** | | | P for trend | ≥60 years | | | P for trend |
| --- | --- | --- | --- | --- | --- | --- | --- | --- |
|  | **T1** | **T2** | **T3** |  | **T1** | **T2** | **T3** |  |
| **Female** |  |  |  |  |  |  |  |  |
| Unadjusted model | 1 | 0.67 (0.11, 4.05) | — | 0.911 | 1 | 0.46 (0.08, 2.78) | 0.71 (0.12, 4.28) | 0.699 |
| Model A^b^ | 1 | Convergence was not attained in 25 iterations | | | 1 | 3.41 (0.03, 346.53) | 3.36 (0.12, 97.86) | 0.741 |
| **Male** |  |  |  |  |  |  |  |  |
| Unadjusted model | 1 | 0.78 (0.07, 8.97) | 1.41 (0.26, 7.81) | 0.840 | 1 | 0.33 (0.04, 3.23) | 0.44 (0.05, 4.22) | 0.566 |
| Model A^b^ | 1 | 1.13 (0.08, 17.02) | 1.68 (0.23, 12.08) | 0.854 | 1 | 0.15 (0.01, 1.87) | 0.19 (0.01, 4.31) | 0.251 |
| Model B^b^ | 1 | Convergence was not attained in 25 iterations | | | 1 | 0.44 (0.01, 16.89) | 2.36 (0.01, 688.54) | 0.729 |

^a^ Values are models adjusted HR and 95% confidence interval, ranges for tertiles (T) 1 through 3. Linear trends (p for trend) were obtained with ratio of fat mass to fat-free mass as continuous variables.

^b^ Model A: adjusted for age, education, smoking, family history of cancer, version of Body Composition Analyzer; Model B: as model A and additionally adjusted for Karnofsky performance scores, NRS 2002 score, nutrition support and handgrip strength;

**Supplementary Table 11.** Association between fat mass to lean body mass ratio and all-cause mortality among patients with normal weight using Cox proportional hazard regression ^a^

|  | **<60 years** | | | P for trend | ≥60 years | | | P for trend |  |
| --- | --- | --- | --- | --- | --- | --- | --- | --- | --- |
|  | **T1** | **T2** | **T3** |  | **T1** | **T2** | **T3** |  |  |
| **Female** |  |  |  |  |  |  |  |  |  |
| Unadjusted model | 1 | 0.92 (0.59, 1.41) | 1.43 (0.82, 2.49) | 0.301 | 1 | 0.72 (0.45, 1.16) | 1.00 (0.60, 1.66) | 0.341 |  |
| Model A^b^ | 1 | 0.93 (0.60, 1.44) | 1.42 (0.81, 2.48) | 0.350 | 1 | 0.75 (0.46, 1.22) | 1.01 (0.60, 1.70) | 0.426 |  |
| Model B^b^ | 1 | 1.15 (0.73, 1.80) | 1.61 (0.90, 2.88) | 0.273 | 1 | 0.75 (0.46, 1.22) | 1.06 (0.62, 1.82) | 0.363 |  |
| Model C^b^ | 1 | 1.09 (0.63, 1.89) | 2.04 (0.94, 4.39) | 0.190 | 1 | 1.01 (0.58, 1.78) | 0.95 (0.50, 1.79) | 0.980 |  |
| Model D^b^ | 1 | 0.85 (0.42, 1.71) | 1.84 (0.62, 5.41) | 0.395 | 1 | 1.48 (0.66, 3.33) | 0.88 (0.35, 2.19) | 0.480 |  |
| **Male** |  |  |  |  |  |  |  |  |  |
| Unadjusted model | 1 | 0.80 (0.54, 1.18) | 0.73 (0.41, 1.28) | 0.382 | 1 | 0.59 (0.42, 0.84) | 0.78 (0.55, 1.12) | 0.012 |  |
| Model A^b^ | 1 | 0.81 (0.54, 1.19) | 0.74 (0.42, 1.32) | 0.425 | 1 | 0.59 (0.42, 0.83) | 0.73 (0.50, 1.06) | 0.010 |  |
| Model B^b^ | 1 | 0.76 (0.51, 1.13) | 0.79 (0.44, 1.40) | 0.366 | 1 | 0.57 (0.40, 0.81) | 0.71 (0.49, 1.03) | 0.007 |  |
| Model C^b^ | 1 | 0.71 (0.42, 1.19) | 0.59 (0.29, 1.23) | 0.241 | 1 | 0.64 (0.43, 0.96) | 0.70 (0.45, 1.08) | 0.047 |  |
| Model D^b^ | 1 | 1.00 (0.51, 1.98) | 0.84 (0.33, 2.13) | 0.924 | 1 | 0.72 (0.42, 1.23) | 0.90 (0.51, 1.58) | 0.472 |  |

^a^ Values are models adjusted HR and 95% confidence interval, ranges for tertiles (T) 1 through 3. Linear trends (p for trend) were obtained with ratio of fat mass to fat-free mass as continuous variables. Normal weight: body mass index 25-<30 kg/m^2^.

^b^ Model A: adjusted for age, education, smoking, alcohol drinking, family history of cancer, version of Body Composition Analyzer; Model B: as model A and additionally adjusted for Karnofsky performance scores, duration of hospital stays, NRS 2002 score, nutrition support and handgrip strength; Model C: as Model B and additionally adjusted for commodities, previous treatments, cancer types, TNM stages, and quality of life; Model D: as Model C and additionally adjusted C-reaction protein.

**Supplementary Table 12.** Baseline characteristics of the current participants with those to follow-up.

|  | Current participants | Participants losing to follow-up | p |
| --- | --- | --- | --- |
| n | 3201 | 1267 |  |
| Female (%) | 1690 (52.80) | 726 (57.30) | 0.005 |
| Age (yrs) | 59.49 (52.54, 65.61) | 62.44 (58.69, 66.61) | 0.057 |
| Quality of life | 46 (42, 52) | 47 (43, 57) | 0.278 |
| **Nutritional indices** |  |  |  |
| Percentage of body fat (%) | 26.45 (20.30, 33.10) | 25.10 (19.20, 30.50) | 0.577 |
| Fat mass (kg) | 16.52 (12.17, 21.11) | 15.12 (11.74, 19.71) | 0.466 |
| Lean body mass(kg) | 42.20 (37.20, 48.70) | 40.70 (37.40, 46.90) | 0.869 |
| RFL | 0.38 (0.27, 0.53) | 0.44 (0.36, 0.60) | 0.068 |
| Body mass index (kg/m^2^) | 23.21 (20.96, 25.24) | 23.88 (20.66, 24.86) | 0.836 |
| Handgrip strength | 23.22 (17.80, 30.75) | 23.93 (18.54, 31.83) | 0.713 |
| C-reactive protein | 3.39 (2.99, 15.30) | 3.23 (0.98, 3.30) | 0.115 |
| PG‐SGA |  |  |  |
| 0-1 | 1507 (47.08) | 59 (62.11) | 0.004 |
| 2-8 | 1420 (44.36) | 34 (35.79) |  |
| ≥9 | 274 (8.56) | 2 (2.11) |  |
| NRS 2002 scores (≥3, %) | 472 (14.75) | 7 (7.37) | 0.040 |
| KPS scores | 90 (90, 90) | 90 (90, 100) | 0.980 |
| Nutrition support ^b^ (yes, %) | 600 (18.74) | 20 (80.00) | 0.902 |
| **Cancer types** |  |  |  |
| Lung cancer | 1088 (33.99) | 30 (31.58) | <0.0001 |
| Breast cancer | 679 (21.21) | 42 (44.21) |  |
| Colorectal cancer | 444 (13.87) | 11 (11.58) |  |
| Esophageal cancer | 77 (2.41) | 0 |  |
| Gastric cancer | 118 (3.69) | 0 |  |
| Others | 795 (24.84) | 12 (12.63) |  |
| **TNM stages** |  |  |  |
| Ⅰ | 310 (9.68) | 9 (12.33) | 0.318 |
| Ⅱ | 446 (13.93) | 7 (9.59) |  |
| Ⅲ | 650 (20.31) | 25 (34.25) |  |
| Ⅳ | 871 (27.21) | 12 (16.44) |  |
| **Treatments** |  |  |  |
| Surgery | 572 (17.87) | 16 (16.84) | 0.076 |
| Chemotherapy | 1905 (59.51) | 15 (15.79) |  |
| Radiotherapy | 78 (2.44) | 58 (61.05) |  |
| Others | 646 (20.18) | 6 (6.32) |  |
| **Duration of hospital stays (days)** | 11 (7, 18) | 11 (7, 18) | 0.928 |
| **Socio-demographics** |  |  |  |
| High education level ^c^ (%) | 2388 (74.60) | 854 (67.37) | 0.100 |
| Smoking (Current, %) | 818 (25.55) | 51 (4.03) | 0.010 |
| Alcohol drinking (yes, %) | 591 (18.46) | 253 (19.97) | 0.859 |
| Tea (yes, %) | 597 (18.65) | 304 (23.99) | 0.497 |
| Family history of cancer (yes, %) | 573 (17.90) | 152 (12.00) | 0.447 |
| Comorbidity (yes, %) | 987 (30.83) | 368 (32.00) | 0.940 |

NOTE: PG-SGA, patient-generated subjective nutrition assessment; NRS 2002, nutrition risk screening 2002; KPS, karnofsky performance scores; RFL: Ratio of fat mass to lean body mass.

^a^ Values are medians (Q1, Q3) or frequencies. Test for difference between tertiles of fat mass to lean body mass ratio was performed by using Kruskal-Wallis tests for non-normally distributed continuous variables and chi-square test for categorical variables.

^b^ Receive enteral nutrition or parenteral nutrition.

^c^ School years ≥12 years.

**Supplementary Table 13.** Association between fat mass to lean body mass ratio and all-cause mortality among participants with BMI < 23.21 kg/m^2^ using Cox proportional hazard regression

|  | **<60 years** | | | p | ≥60 years | | | p |
| --- | --- | --- | --- | --- | --- | --- | --- | --- |
|  | **T1** | **T2** | **T3** |  | **T1** | **T2** | **T3** |  |
| **Female** |  |  |  |  |  |  |  |  |
| Unadjusted model | 1 | 0.99 (0.61, 1.60) | 0.97 (0.35, 2.69) | 0.998 | 1 | 0.67 (0.40, 1.11) | 1.14 (0.57, 2.25) | 0.226 |
| Model A^b^ | 1 | 1.06 (0.65, 1.73) | 1.05 (0.38, 2.93) | 0.970 | 1 | 0.69 (0.41, 1.16) | 1.16 (0.58, 2.31) | 0.281 |
| Model B^b^ | 1 | 1.29 (0.77, 2.14) | 1.14 (0.40, 3.26) | 0.626 | 1 | 0.72 (0.42, 1.23) | 1.19 (0.58, 2.45) | 0.339 |
| Model C^b^ | 1 | 1.18 (0.59, 2.35) | 2.00 (0.61, 6.51) | 0.497 | 1 | 0.80 (0.43, 1.48) | 1.01 (0.41, 2.52) | 0.754 |
| Model D^b^ | 1 | 0.74 (0.27, 2.08) | 9.13 (1.22, 68.37) | 0.077 | 1 | 1.65 (0.27, 1.54) | 0.49 (0.10, 2.40) | 0.453 |
| **Male** |  |  |  |  |  |  |  |  |
| Unadjusted model | 1 | 0.86 (0.54, 1.37) | 0.42 (0.13, 1.33) | 0.301 | 1 | 0.62 (0.43, 0.90) | 0.87 (0.57, 1.33) | 0.043 |
| Model A^b^ | 1 | 0.86 (0.54, 1.38) | 0.42 (0.13, 1.36) | 0.321 | 1 | 0.62 (0.42, 0.90) | 0.79 (0.51, 1.23) | 0.039 |
| Model B^b^ | 1 | 0.82 (0.50, 1.33) | 0.50 (0.15, 1.64) | 0.419 | 1 | 0.61 (0.42, 0.89) | 0.76 (0.48, 1.21) | 0.036 |
| Model C^b^ | 1 | 0.81 (0.41, 1.59) | 0.63 (0.18, 2.13) | 0.661 | 1 | 0.63 (0.41, 0.99) | 0.73 (0.42, 1.28) | 0.112 |
| Model D^b^ | 1 | 0.83 (0.32, 2.17) | 2.26 (0.52, 9.81) | 0.452 | 1 | 0.71 (0.39, 1.28) | 1.09 (0.51, 2.29) | 0.429 |

^a^ Values are models adjusted HR and 95% confidence interval, ranges for tertiles (T) 1 through 3. Linear trends (p for trend) were obtained with lean body mass as continuous variables.

^b^ Model A: adjusted for age, education, smoking, alcohol, family history of cancer, version of Body Composition Analyzer; Model B: as model A and additionally adjusted for Karnofsky performance scores, duration of hospital stays, NRS 2002 score, nutrition support and handgrip strength; Model C: as Model B and additionally adjusted for commodities, previous treatments, cancer types TNM stages, and quality of life; Model D: as Model C and additionally adjusted C-reaction protein.

**Supplementary Table 14.** Association between fat mass to lean body mass ratio and all-cause mortality among participants with BMI ≥ 23.21 kg/m^2^ using Cox proportional hazard regression

|  | **<60 years** | | | p | ≥60 years | | | p |
| --- | --- | --- | --- | --- | --- | --- | --- | --- |
|  | **T1** | **T2** | **T3** |  | **T1** | **T2** | **T3** |  |
| **Female** |  |  |  |  |  |  |  |  |
| Unadjusted model | 1 | 0.75 (0.38, 1.47) | 0.84 (0.45, 1.59) | 0.705 | 1 | 1.64 (0.58, 4.67) | 1.17 (0.42, 3.25) | 0.295 |
| Model A^b^ | 1 | 0.80 (0.40, 1.57) | 0.81 (0.43, 1.52) | 0.773 | 1 | 2.02 (0.69, 5.91) | 1.31 (0.46, 3.72) | 0.150 |
| Model B^b^ | 1 | 0.91 (0.46, 1.82) | 0.87 (0.46, 1.67) | 0.919 | 1 | 1.78 (0.60, 5.23) | 1.15 (0.40, 3.32) | 0.174 |
| Model C^b^ | 1 | 0.94 (0.40, 2.22) | 0.92 (0.42, 2.03) | 0.978 | 1 | 1.69 (0.51, 5.61) | 1.13 (0.34, 3.71) | 0.306 |
| Model D^b^ | 1 | 1.13 (0.26, 4.97) | 0.42 (0.11, 1.65) | 0.164 | 1 | 71.82 (3.35, 1541.10) | 18.38 (0.84, 403.56) | 0.001 |
| **Male** |  |  |  |  |  |  |  |  |
| Unadjusted model | 1 | 0.81 (0.42, 1.55) | 0.92 (0.50, 1.71) | 0.770 | 1 | 0.36 (0.17, 0.77) | 0.40 (0.20, 0.81) | 0.024 |
| Model A^b^ | 1 | 0.72 (0.37, 1.40) | 0.83 (0.44, 1.55) | 0.613 | 1 | 0.36 (0.17, 0.78) | 0.38 (0.18, 0.79) | 0.025 |
| Model B^b^ | 1 | 0.77 (0.39, 1.50) | 0.78 (0.41, 1.50) | 0.720 | 1 | 0.34 (0.15, 0.73) | 0.36 (0.17, 0.76) | 0.017 |
| Model C^b^ | 1 | 0.74 (0.31, 1.77) | 0.82 (0.37, 1.84) | 0.791 | 1 | 0.43 (0.15, 1.23) | 0.50 (0.18, 1.39) | 0.288 |
| Model D^b^ | 1 | 0.84 (0.29, 2.49) | 0.81 (0.31, 2.14) | 0.912 | 1 | 0.33 (0.10, 1.11) | 0.61 (0.20, 1.88) | 0.115 |

^a^ Values are models adjusted HR and 95% confidence interval, ranges for tertiles (T) 1 through 3. Linear trends (p for trend) were obtained with lean body mass as continuous variables.

^b^ Model A: adjusted for age, education, smoking, alcohol, family history of cancer, version of Body Composition Analyzer; Model B: as model A and additionally adjusted for Karnofsky performance scores, duration of hospital stays, NRS 2002 score, nutrition support and handgrip strength; Model C: as Model B and additionally adjusted for commodities, previous treatments, cancer types TNM stages, and quality of life; Model D: as Model C and additionally adjusted C-reaction protein.

**Supplementary Table 15.** Association between fat mass to lean body mass ratio and all-cause mortality using Cox proportional hazard regression (n=3358)

|  | **<60 years** | | | p | ≥60 years | | | p |
| --- | --- | --- | --- | --- | --- | --- | --- | --- |
|  | **T1** | **T2** | **T3** |  | **T1** | **T2** | **T3** |  |
| **Female** |  |  |  |  |  |  |  |  |
| Unadjusted model | 1 | 0.731 (0.50, 1.06) | 0.66 (0.45, 0.97) | 0.077 | 1 | 0.82 (0.55, 1.22) | 0.77 (0.53, 1.11) | 0.363 |
| Model A^b^ | 1 | 0.75 (0.52, 1.10) | 0.63 (0.43, 0.94) | 0.061 | 1 | 0.94 (0.62, 1.41) | 0.86 (0.59, 1.27) | 0.743 |
| Model B^b^ | 1 | 0.94 (0.64, 1.39) | 0.74 (0.49, 1.11) | 0.322 | 1 | 1.06 (0.70, 1.61) | 0.98 (0.65, 1.46) | 0.915 |
| Model C^b^ | 1 | 1.05 (0.66, 1.67) | 0.82 (0.49, 1.37) | 0.632 | 1 | 1.30 (0.81, 2.08) | 1.14 (0.71, 1.83) | 0.555 |
| Model D^b^ | 1 | 0.85 (0.47, 1.54) | 0.60 (0.30, 1.20) | 0.354 | 1 | 2.19 (1.10, 4.34) | 1.02 (0.50, 2.05) | 0.026 |
| Model C+BMI | 1 | 1.22 (0.75, 2.00) | 1.19 (0.62, 2.29) | 0.723 |  | 1.17 (0.70, 1.95) | 0.93 (0.50, 1.71) | 0.585 |
| Model C+BMI+CRP |  | 0.94 (0.48, 1.81) | 0.74 (0.29, 1.87) | 0.811 |  | 1.85 (0.89, 3.87) | 0.73 (0.30, 1.77) | 0.018 |
| **Male** |  |  |  |  |  |  |  |  |
| Unadjusted model | 1 | 0.80 (0.56, 1.14) | 0.79 (0.56, 1.14) | 0.339 | 1 | 0.59 (0.43, 0.80) | 0.57 (0.43, 0.77) | 0.000 |
| Model A^b^ | 1 | 0.81 (0.56, 1.16) | 0.82 (0.57, 1.18) | 0.429 | 1 | 0.60 (0.44, 0.82) | 0.56 (0.42, 0.75) | 0.000 |
| Model B^b^ | 1 | 0.82 (0.57, 1.19) | 0.92 (0.63, 1.34) | 0.589 | 1 | 0.59 (0.43, 0.81) | 0.56 (0.42, 0.76) | 0.000 |
| Model C^b^ | 1 | 0.81 (0.50, 1.30) | 0.95 (0.61, 1.49) | 0.665 | 1 | 0.65 (0.45, 0.95) | 0.65 (0.45, 0.92) | 0.028 |
| Model D^b^ | 1 | 0.932 (0.50, 1.74) | 1.18 (0.68, 2.07) | 0.720 | 1 | 0.76 (0.47, 1.22) | 0.80 (0.51, 1.26) | 0.486 |
| Model C+BMI | 1 | 0.74 (0.45, 1.22) | 0.75 (0.40, 1.38) | 0.463 |  | 0.69 (0.46, 1.02) | 0.71 (0.45, 1.11) | 0.160 |
| Model C+BMI+CRP | 1 | 0.86 (0.45, 1.63) | 0.92 (0.44, 1.93) | 0.900 |  | 0.78 (0.47, 1.30) | 0.85 (0.48, 1.50) | 0.645 |

^a^ Values are models adjusted HR and 95% confidence interval, ranges for tertiles (T) 1 through 3. Linear trends (p for trend) were obtained with lean body mass as continuous variables.

^b^ Model A: adjusted for age, education, smoking, alcohol, family history of cancer, version of Body Composition Analyzer; Model B: as model A and additionally adjusted for Karnofsky performance scores, duration of hospital stays, NRS 2002 score, nutrition support and handgrip strength; Model C: as Model B and additionally adjusted for commodities, previous treatments, cancer types TNM stages, and quality of life; Model D: as Model C and additionally adjusted C-reaction protein.
